# Arene guest selectivity and pore flexibility in a metal–organic framework with semi-fluorinated channel walls

**DOI:** 10.1098/rsta.2016.0031

**Published:** 2017-01-13

**Authors:** Rebecca Smith, Iñigo J. Vitórica-Yrezábal, Adrian Hill, Lee Brammer

**Affiliations:** 1 Department of Chemistry, University of Sheffield, Brook Hill, Sheffield S3 7HF, UK; 2 Johnson Matthey Process Technologies, Inc., Savannah, GA 31408, USA

**Keywords:** porous materials, coordination chemistry, metal–organic framework, solid-state transformation, *in situ* powder diffraction, solid-state NMR

## Abstract

A metal–organic framework (MOF) with one-dimensional channels of approximately hexagonal cross-section [Ag_2_(O_2_CCF_2_CF_2_CO_2_)(TMP)] **1** (TMP =2,3,5,6-tetramethylpyrazine) has been synthesized with MeOH filling the channels in its as-synthesized form as [Ag_2_(O_2_CCF_2_CF_2_CO_2_)(TMP)]·*n*(MeOH) **1-MeOH** (*n* = 1.625 by X-ray crystallography). The two types of ligand connect columns of Ag(I) centres in an alternating manner, both around the channels and along their length, leading to an alternating arrangement of hydrocarbon (C–H) and fluorocarbon (C–F) groups lining the channel walls, with the former groups projecting further into the channel than the latter. MeOH solvent in the channels can be exchanged for a variety of arene guests, ranging from xylenes to tetrafluorobenzene, as confirmed by gas chromatography, ^1^H nuclear magnetic resonance (NMR) spectroscopy, thermogravimetric analysis and ^13^C cross-polarization magic angle spinning NMR spectroscopy. Alkane and perfluoroalkane guests, however, do not enter the channels. Although exhibiting some stability under a nitrogen atmosphere, sufficient to enable crystal structure determination, the evacuated MOF **1** is unstable for periods of more than minutes under ambient conditions or upon heating, whereupon it undergoes an irreversible solid-state transformation to a non-porous polymorph **2**, which comprises Ag_2_(O_2_CCF_2_CF_2_CO_2_) coordination layers that are pillared by TMP ligands. This transformation has been followed *in situ* by powder X-ray diffraction and shown to proceed via a crystalline intermediate.

This article is part of the themed issue ‘Coordination polymers and metal–organic frameworks: materials by design’.

## 1. Introduction

Coordination polymers, and particularly metal–organic frameworks (MOFs), which are the focus of this theme issue, have become an intensely studied field of research over the past 20–25 years [1–3]. The interest stems from the modular design of these materials, and the potential thereby to tune physical and chemical properties and potentially to design rather than discover materials with high performance for a particular purpose. Applications of MOFs (also known as porous coordination polymers, PCPs) depend on their porosity, and include adsorption and separation of gases and other small molecules, sensing, catalysis, magnetic and conduction behaviour and drug delivery [4–11]. Although many MOFs form quite rigid networks, which may exhibit porosity on guest removal from the pores, there is an increasing interest in developing and understanding MOFs that exhibit flexibility and therefore may be able to respond to external stimuli (temperature, pressure, light, etc.) by changing the shape and size of their pores and therefore their physical properties [12–15].

**Scheme 1.**
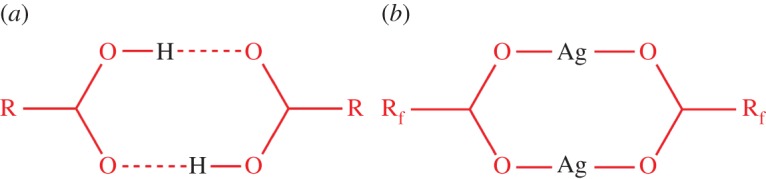
Structural analogy between (*a*) hydrogen-bonded carboxylic acid dimer (R = alkyl, aryl group) and (*b*) silver carboxylate dimer (R_f_ = perfluoroalkyl group).

**Scheme 2.**
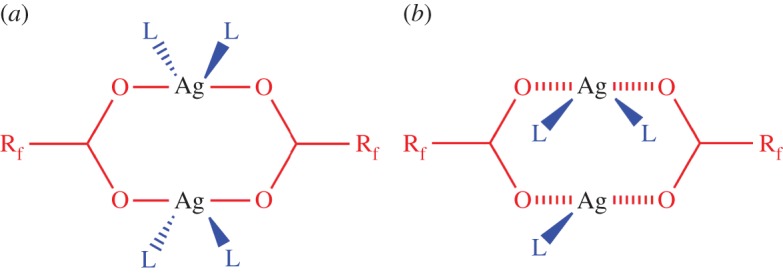
Examples of silver(I) perfluorocarboxylate dimer secondary building units, connected by neutral (ditopic) diimine ligands, L, to propagate coordination polymers.

For a number of years, we have been exploring the behaviour of families of coordination polymers consisting of Ag(I) centres linked by perfluoroalkyl carboxylates and diimine or diamine ligands. The impetus for this avenue of research was the recognition of the structural analogy between dimer motif Ag_2_(O_2_CR_f_)_2_ and the hydrogen-bonded 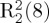 dimer commonly formed by pairs of carboxylic acids (scheme 1) [16,17]. Our work to date has concentrated on one-dimensional coordination polymers and two-dimensional layered materials formed by linking of the Ag_2_(O_2_CR_f_)_2_ dimer units or similar building blocks via diimine ligands (scheme 2). The coordination polymers, although often not porous in a conventional sense, have been shown to encapsulate small molecules from the vapour phase (e.g. alcohols, arenes), often without loss of crystallinity in the materials [18–22]. These processes, often followed by *in situ* diffraction or spectroscopic studies, are enabled by mobility of the fluoralkyl chains of the carboxylate ligands and the flexibility in coordination at the Ag(I) centres, which takes advantage of the lack of strong preferences in coordination geometry of the d^10^ metal centres and the lability of the metal–ligand bonds [20].

Building on our original inspiration from the analogy between Ag–O coordination bonding and hydrogen bonding, we have sought further exploration of the architectures that can be developed by linking silver(I) perfluoroalkylcarboxylate building blocks and taking advantage of their flexibility. In particular, we have considered the wide range of crystalline architectures that have been developed and designed through hydrogen bonding between guanidinium and sulfonate ions in the extensive body of work reported by Ward and co-workers [23–27]. These materials were initially developed as hydrogen-bonded layers or as three-dimensional frameworks assembled around guest molecules [23,24], but the flexibility of the hydrogen bonding interaction common to the entire family of materials was later exploited to develop both cylindrical (channel) [26] and polyhedral (cage) assemblies [27].

In this study, we report the development of a cylindrical channel structure built from silver(I) centres linked by perfluoroalkylcarboxylate and diimine linkers. The structure of [Ag_2_(O_2_CCF_2_CF_2_CO_2_)(TMP)]·*n*(MeOH) **1-MeOH** comprises columns of Ag(I) centres linked via the tetrafluorosuccinate and tetramethylpyrazine (TMP) ligands, leading to a framework which exhibits an array of parallel hexagonal channels that contain MeOH solvent molecules (*n* = 1.625 modelled from the single-crystal structure) in the as-synthesized material. We show that the MeOH molecules can be exchanged for a variety of aromatic guests whose presence has been identified by ^1^H nuclear magnetic resonance (NMR) spectroscopy and solid-state ^13^C NMR spectroscopy, gas chromatography (GC) and thermogravimetric analysis (TGA). By contrast, aliphatic guests are not included in the channels. We further show that the MeOH molecules can be removed to reveal an empty channel material, which upon heating can be collapsed to a new condensed phase, the transition to which has been followed *in situ* by powder X-ray diffraction (PXRD).

## 2. Experimental set-up

### (a) General

Silver carbonate (99%), tetramethylpyrazine (98%) perfluoro(methylcyclohexane) (90%) and tetramethylbenzene (98%) were purchased from Sigma Aldrich. Tetrafluorosuccinic acid (98%) and tetrafluorobenzene (98%) were purchased from Alfa Aesar. High-performance liquid chromatography-grade solvents methanol, toluene, pentane, cyclohexane, *o*-xylene, *m*-xylene and *p*-xylene were purchased from VWR. All chemicals were used as received. Elemental analysis was carried out by the University of Sheffield Department of Chemistry elemental analysis service, using a Perkin-Elmer 2400 CHNS/O Series II Elemental Analyser.

### (b) Synthesis

#### (i) [Ag_2_(O_2_CCF_2_CF_2_CO_2_)(TMP)]·*n*(MeOH) **1-MeOH**

**Figure 1.**
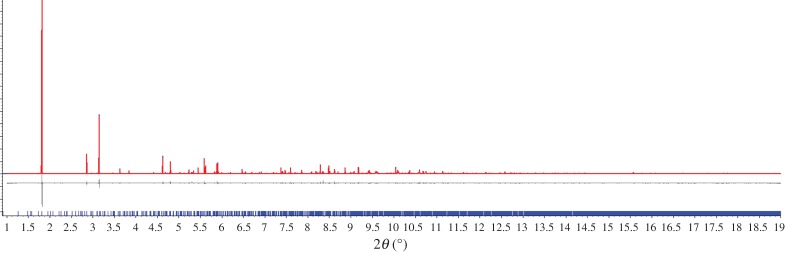
Observed (blue) and calculated (red) profiles and difference plot [(*I*_obs _− *I*_calcd_)] (grey) at 298 K of the Pawley refinement for **1-MeOH** (1.0 ≤ 2*θ* ≤ 19.0°, *d*_min_ = 1.22 Å); *R*_wp_ = 0.162; *R*_wp_′ = 0.200.

Ag_2_CO_3_ (15 mg, 0.05 mmol) in methanol (15 ml) was sonicated for 15 min. This solution was added to a solution of TMP (15 mg, 0.11 mmol) in methanol (2 ml). A solution of tetrafluorosuccinic acid (41 mg, 0.22 mmol) in methanol (2 ml) was then added. Slow evaporation of methanol for 48 h at room temperature yielded white crystalline [Ag_2_(O_2_CCF_2_CF_2_CO_2_)(TMP)]·*n*(MeOH) (**1-MeOH**). Yield 68% (20 mg, 0.034 mmol). Anal. found: C, 26.56%; H, 2.08%; N, 5.06%; calcd C, 26.79%; H, 2.25%; N, 5.21% (for *n* = 0 as MeOH is easily lost from crystals prior to analysis). Phase purity was confirmed by PXRD (figure 1). Single crystals suitable for X-ray diffraction were used for crystal structure determination.

#### (ii) [Ag_2_(O_2_CCF_2_CF_2_CO_2_)(TMP)] **1**

Crystals of **1-MeOH** were left to dry on a glass slide at room temperature. After 5 min under ambient conditions, the colourless crystals of **1-MeOH** became slightly opaque upon loss of the MeOH molecules and formation of the empty framework [Ag_2_(O_2_CCF_2_CF_2_CO_2_)(TMP)] **1** initially as single crystals, which were used for characterization by single-crystal X-ray diffraction under a nitrogen atmosphere. Under ambient conditions **1** converts over a period of minutes into its more stable non-porous polymorph, **2**, as a polycrystalline solid.

#### (iii) [Ag_2_(O_2_CCF_2_CF_2_CO_2_)(TMP)] **2**

Ag_2_CO_3_ (15 mg, 0.05 mmol) in acetonitrile (15 ml) was sonicated for 15 min. This solution was added to a solution of 2,3,5,6-TMP (15 mg, 0.11 mmol) in dichloromethane (1 ml). A solution of tetrafluorosuccinic acid (41 mg, 0.22 mmol) in acetonitrile (2 ml) was then added. Slow evaporation of solvent for 72 h at room temperature yielded white crystalline [Ag_2_(O_2_CCF_2_CF_2_CO_2_)(TMP)] **2**. Yield 55% (30 mg). Calcd. C, 26.7; H, 2.20; N, 5.20%; found C, 26.44; H, 2.01; N, 4.97%. Single crystals suitable for X-ray diffraction were used for crystal structure determination.

### (c) Single-crystal X-ray diffraction

X-ray data were collected for compounds [Ag_2_(O_2_CCF_2_CF_2_CO_2_)(TMP)]·*n*(MeOH) **1-MeOH** at 150 K, [Ag_2_(O_2_CCF_2_CF_2_CO_2_)(TMP)] **1** at 298 K and [Ag_2_(O_2_CCF_2_CF_2_CO_2_)(TMP)] **2** at 150 K using Mo-K_α_ radiation on a Bruker APEX II CCD diffractometer equipped with an Oxford Cryosystems Cobra Plus nitrogen flow gas system. Data reduction was carried out with Bruker APEX2 or Rigaku-Oxford Diffraction CrysAlisPro programs. Absorption correction was performed using empirical methods based upon symmetry-equivalent reflections combined with measurements at different azimuthal angles using either the program SADABS [28,29]^1^ or SCALE3 ABSPACK. The crystal structures were solved and refined against all *F*^2^ values using the SHELXL [30] or Olex2 [31] programs. All non-hydrogen atoms were refined anisotropically with the exception of the methanol solvent molecules in the **1-MeOH** structure. Hydrogen atoms were placed in calculated positions refined using idealized geometries (riding model) and assigned fixed isotropic displacement parameters. The C–O distances of the methanol molecules in **1-MeOH** were restrained to be similar in length (SADI and DFIX commands in the SHELXL program). Rigid bond restraints and similarity restraints between neighbouring atoms were applied to the displacement parameters of the ligand atoms in **1-MeOH** (RIGU and SIMU commands in SHELXL program). The TwinRotMat protocol in the program PLATON [32] was used to determine the twin law (inversion twin 0.5077(7):0.4989(7)) for the crystal structure of compound **1-MeOH**. A summary of crystallographic data is provided in table 1.

**Table 1.** Data collection, structure solution and refinement parameters for crystal structures of **1-MeOH**, **1** and **2**.

	**1-MeOH**	**1**	**2**
crystal colour	colourless	colourless	colourless
crystal size (mm)	0.6 × 0.3 × 0.3	0.70 × 0.07 × 0.07	0.35 × 0.20 × 0.02
crystal system	rhombohedral	rhombohedral	monoclinic
space group, *Z*	*R* 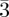 , 144	*R* 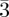 *c*, 36	*P*2_1_/*n*, 2
*a* (Å)	50.0474 (6)	25.1849 (4)	10.5304 (10)
*b* (Å)	50.0474 (6)	25.1849 (4)	5.6409 (5)
*c* (Å)	32.7348 (4)	33.2733 (7)	12.1404 (10)
*β* (°)	90	90	90.863 (6)
*V* (Å^3^)	71 007.5 (18)	18 277.0 (7)	721.07 (11)
density (Mg m^−3^)	1.972	1.766	2.487
wavelength (Å)	0.71073	0.71073	0.71073
temperature (K)	150	298	100
*μ*(Mo-Kα) (mm^−1^)	2.051	1.978	2.785
2*θ* range (°)	3.52–50.70	4.467–50.70	5.08–55.17
reflns collected	115 207	98 053	11 467
independent reflns (*R*_int_)	28 859 (0.0585)	3728 (0.0477)	1665 (0.0396)
reflns used in refinement, *n*	28 859	3728	1665
L.S. parameters, *p*	1862	223	111
restraints, *r*	747	14	0
*R*1(*F*)^a^ *I* > 2.0*σ(I)*	0.0564	0.0276	0.0215
w*R*^2^(*F*^2^),^b^ all data	0.1142	0.0757	0.0512
*S*(*F*^2^),^c^ all data	1.128	1.070	1.067

^a^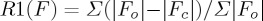; ^b^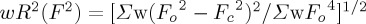; ^c^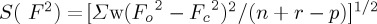.

### (d) Powder X-ray diffraction

A sample of **1-MeOH** was loaded into a 0.7 mm borosilicate capillary and data were collected (*λ* = 0.3999(7) Å) at beamline ID31 (now ID22) [33] at the European Synchrotron Radiation Facility (ESRF) using a nine-channel multi-analyser crystal (MAC) detector. Four scans were collected at a scan speed of 6° min^−1^ in the range −2.5 ≤ 2*θ* ≤ 12°, during which the capillary was spinning. The diffraction patterns were merged, and the combined pattern was indexed and fitted using the TOPAS Academic program [34], by Pawley refinement [35] for data with *d*_min_ ≤ 1.22 Å, using a starting unit cell model from the single-crystal structure determination (figure 1).

**Figure 2.**
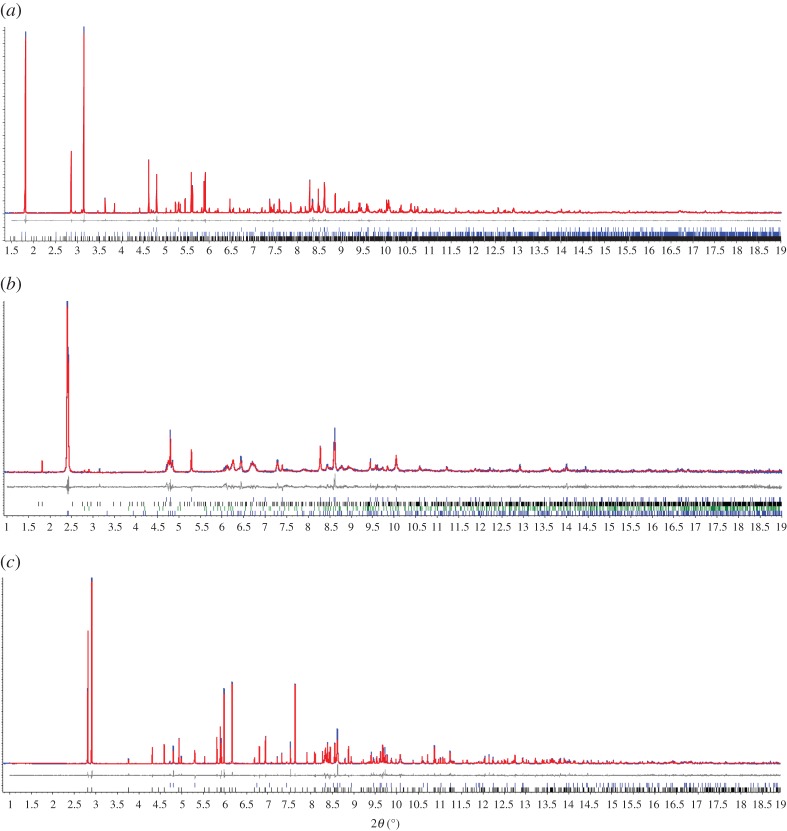
Observed (blue) and calculated (red) profiles and difference plot [(*I*_obs _− *I*_calcd_)] (grey) at 298 K (1.0 ≤ 2*θ* ≤ 19.0°, *d*_min_ = 1.22 Å) of (*a*) the joint Pawley refinement for **1-MeOH** and **1** and Rietveld refinement [37] for Ag_2_CO_3_ (*R*_wp_ = 0.081; *R*_wp_′ = 0.125); (*b*) the Pawley refinement of the intermediate phase (see below) and Rietveld refinement for **1, 2** and Ag_2_CO_3_ (*R*_wp_ = 0.181; *R*_wp_′ = 0.308); (*c*) Rietveld refinement of **2** and Ag_2_CO_3_ (*R*_wp_ = 0.194; *R*_wp_′ = 0.270).

The transformation of microcrystalline **1-MeOH** into **2** was monitored *in situ* by PXRD (*λ* = 0.3999(7) Å) at beamline ID31 (now ID22) [33] at the ESRF using a nine-channel MAC detector. A sample of colourless microcrystalline **1-MeOH** was synthesized as previously described. Colourless crystals of **1-MeOH** were ground in the mother liquor with a glass rod and introduced into a 1 mm kapton capillary. The 1 mm kapton capillary was introduced into a 1.5 mm quartz capillary where the microcrystalline sample was unloaded with the help of a fine glass rod. Finally, the kapton capillary was removed before conducting the diffraction experiment. Five initial scans were collected at a scan speed of 6° min^−1^ in the range −2.5 ≤ 2*θ* ≤ 12°, during which the capillary was spinning (note: a larger range of 2*θ* is accessed due to the angular range of the nine-channel detector). These patterns were merged and used to check the phase purity of the starting material, establishing by Pawley fitting that both compound **1-MeOH** and **1** are present and that the sample contains a very small amount of Ag_2_CO_3_ starting material as an impurity (figure 2*a*) [36]. The sample was then heated to 323 K for 18 min, during which six patterns were measured, but indicated no changes. The temperature was then increased to 373 K, after which three patterns were measure in a 9 min period, and cooled to 298 K, after which a further three patterns were measured in a 9 min period. Fitting for the first pattern obtained at 373 K, which allowed indexing of the intermediate phase, and the final pattern at 298 K, which confirms full conversion to **2**, is shown in figure 2*b,c*, respectively.

### (e) NMR spectroscopy

^1^H NMR spectra were recorded on a Bruker 400 MHz Fourier transform NMR (FT-NMR) spectrometer. Samples of **1-MeOH** (approx. 2–5 mg) after soaking in different solvent guests were dissolved in dimethyl sulfoxide (DMSO)-d6 before analysis. Chemical shifts are reported in ppm relative to tetramethylsilane (0 ppm) with the residual solvent resonance as an internal standard. The NMR data were analysed using the Bruker Topspin program (see electronic supplementary material, tables S1–S9).

^13^C cross-polarization magic angle spinning (CP-MAS) NMR spectra (spinning speed 8–10 Hz) were recorded on a Bruker 500 MHz FT-NMR spectrometer (125.669 MHz for ^13^C). Data were processed using Bruker TOPSPIN v. 1.3 and MestRe Nova software (see electronic supplementary material, figures S8–S13). All chemical shifts were referenced to a solid sample of adamantane.

### (f) Thermogravimetric analysis

TGA was conducted using a Perkin-Elmer Pyris1 TGA model thermogravimetric analyser. Samples were heated from 25°C to 450°C at 5°C min^−1^, under a nitrogen atmosphere (see electronic supplementary material, figures S3–S7).

### (g) Gas chromatography

Samples of **1-MeOH** (approx. 2–5 mg) were soaked in different solvent guests and dried in air for 5 min before dissolving in DMSO. The solutions were transferred to screw-cap glass vials and analysed using a Perkin-Elmer Autosystem GC with an Alltech^TM^ Heliflex^TM^ AT-1 capillary column (L × I.D. 30 m × 0.32 mm × d.f. 5.00 µm), heating from 40°C to 200°C at 10°C min^−1^. Expected guest retention times were determined from DMSO solutions of each guest and found to be 2.2 min (methanol), 3.6 min (perfluoro(methylcyclohexane)), 9.6 min (1,2,4,5-tetrafluorobenzene), 10.3 min (cyclohexane), 11.39 min (pentane), 12.8 min (toluene), 15.1 min (*p*-xylene), 15.2 min (*m*-xylene) and 15.6 min (*o*-xylene). The relative content of guests was determined by comparing peak areas with that of TMP (retention time 18.9 min). The gas chromatograms can be found in the electronic supplementary material, figures S15–S22.

## 3. Results

### (a) Synthesis and crystal structures of **1-MeOH** and **1**

Synthesis by slow evaporation of MeOH from a solution of the reagents yielded crystalline **1-MeOH** in 68% yield as a phase-pure material established by Pawley refinement of its X-ray powder pattern. In some repetitions of the synthesis, residual undissolved starting material Ag_2_CO_3_ remained present in small amount (see *in situ* PXRD study, see below).

**Figure 3.**
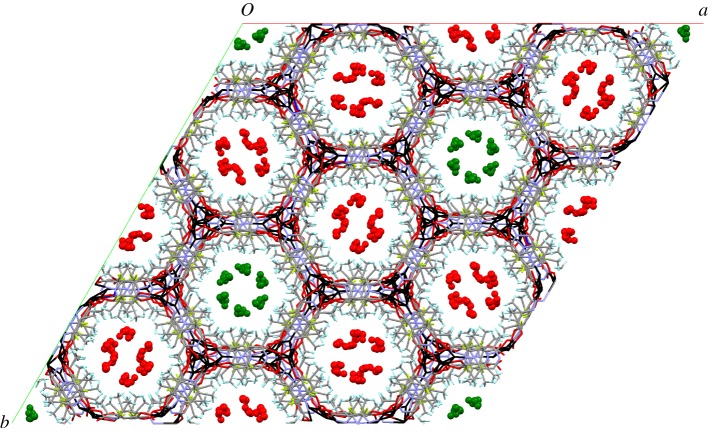
Crystal structure of **1-MeOH** viewed down the *c*-axis showing the two types of hexagonal channels containing MeOH molecules (A channels, MeOH in green; B channels, MeOH in red). Silver atoms are in black, carbon in grey, hydrogen in cyan, nitrogen in blue, fluorine in yellow.

**Figure 4.**
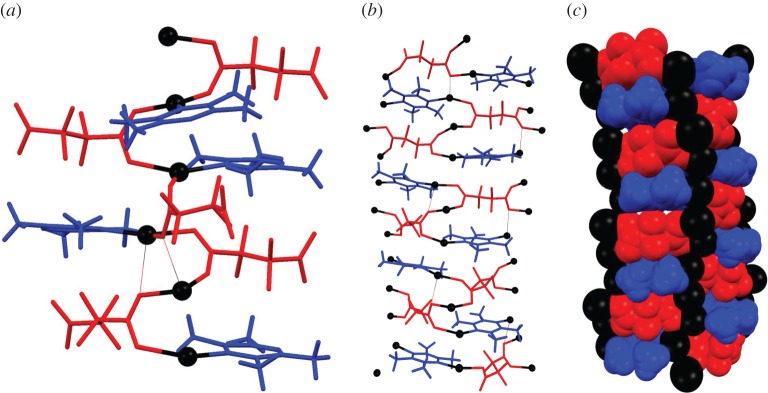
(*a*) A section of the column of Ag(I) centres that lies along the intersection of channel walls (along the *c*-axis) in **1-MeOH** showing the trigonal (and occasional pseudotetrahedral) coordination of Ag(I) centres. (*b*) Section showing two walls of a channel emphasizing the alternating arrangement of ligands. (*c*) Same section as shown in (*b*), but in space-filling representation to illustrate the rugose channel wall surfaces. Long Ag–O bonds 2.54 < Ag–O < 2.68 Å are shown as thin lines. Ag atoms are in black, tetrafluorosuccinate ligands in red, TMP ligands in blue.

The crystal structure of **1-MeOH** shows that it exhibits a framework structure with one-dimensional hexagonal channels that run along the *c*-axis (figure 3). There are two crystallographically distinct channels, A and B, of the same dimensions with both containing sites of inversion symmetry and one also containing 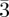 symmetry axes (B) parallel to the channels. The channels contain MeOH molecules in the as-synthesized form. The framework is constructed from columns of Ag(I) centres that are coordinated in a trigonal manner by oxygen atoms from two tetrafluorosuccinate ligands and nitrogen from one TMP ligand (figure 4). The carboxylate groups of the former bridge between pairs of Ag(I) centres along the chain via coordination of their *syn* lone pairs (2.23 < Ag–O < 2.38 Å), and in some case form longer bridging interactions via an *anti*-lone pair to a third Ag(I) centre in the column (2.54 < Ag–O < 2.68 Å), rendering some Ag(I) centres pseudo-tetrahedral in coordination. Ag–N distances lie in the range 2.210(9)–2.244(9) Å. Ag(I) centres are separated by distances in the range 2.965(1)–3.355(1) Å along these columns. The walls of the channels are formed by the two types of ligands, which bridge between Ag(I) centres in neighbouring columns and are present in an alternating arrangement upon descending the channels (figure 4). The TMP ligands are oriented approximately orthogonal to the channel axis (*c*-axis), thereby projecting their methyl groups into the channel and leading to a rugose surface of alternating alkyl and fluoroalkyl groups down the channel walls. Crystals of **1-MeOH** lose methanol very easily, even at room temperature, as indicated initially by elemental analysis. The best estimate of the methanol content comes from the single-crystal structure determination, in which 13 MeOH molecules are identified and refined to give a composition of the asymmetric unit of [Ag_16_(O_2_CCF_2_CF_2_CO_2_)_8_(TMP)_8_]·13(MeOH), which simplifies to the reported formula of [Ag_2_(O_2_CCF_2_CF_2_CO_2_)(TMP)]·1.625(MeOH). It is likely, however, that this is a slight underestimate of the maximum attainable MeOH content of the channels.

**Figure 5.**
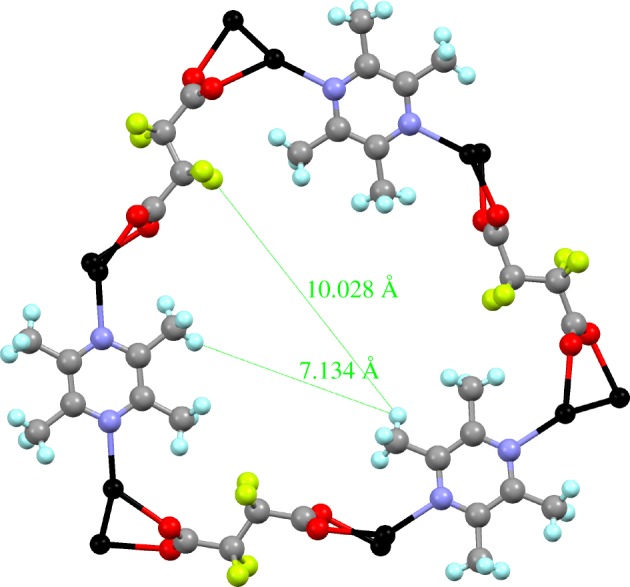
Crystal structure of **1** viewed down the *c*-axis showing one ring that defines the channels and its dimensions. Colours as in figure 3.

Upon allowing a crystal of **1-MeOH** to stand in air for 5 min the MeOH in the channels is lost, but the crystallinity is retained allowing a single-crystal structure determination of **1** (see electronic supplementary material, figures S1 and S2). The desolvation was repeated a number of times and is reproducible. The crystal structure of **1** retains the framework already described, albeit with a 1.6% expansion along the channel direction (*c*-axis), but almost no change in channel width, which retains dimensions in the range 7–10 Å between atoms that lie across the channel (figure 5). The crystal structure of **1** is of higher symmetry than **1-MeOH**, although still rhombohedral, having undergone approximately halving of the *a*- and *b*-axis lengths and a change in the space group from *R*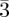 to *R*
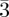
*c*. Most noticeable is that the composition of the asymmetric unit comprising [Ag_16_(O_2_CCF_2_CF_2_CO_2_)_8_(TMP)_8_]·13(MeOH) in **1-MeOH** is reduced to [Ag_2_(O_2_CCF_2_CF_2_CO_2_)(TMP)] in **1**.

**Figure 6.**
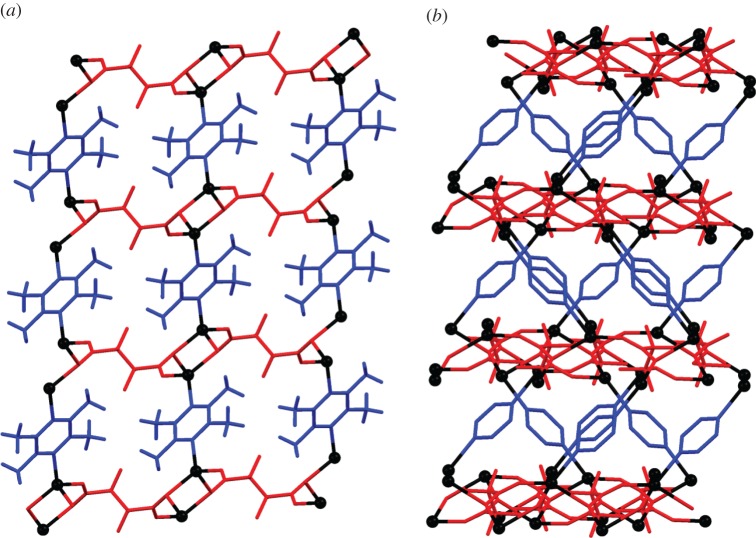
Crystal structure of **2** viewed (*a*) down the *b*-axis and (*b*) along the (101) direction showing Ag(O_2_CCF_2_CF_2_CO_2_) layers pillared by TMP ligands. Methyl groups have been removed for clarity in (*b*). Colours as in figure 4.

An analogous synthetic procedure to that used to prepare **1-MeOH**, but using MeCN and CH_2_Cl_2_ as solvents instead of MeOH, yielded crystals of **2**, which has the same composition as but different crystal structure from that of **1**. The structure of **2** includes no channels or open pore space (figure 6), but rather consists of layers of composition Ag(O_2_CCF_2_CF_2_CO_2_) which are pillared by TMP ligands that are inclined to the planes of these layers.

### (b) Guest selectivity

Having established that MeOH can be removed from the channels of **1-MeOH** to give **1**, a series of experiments were conducted to establish the potential for framework **1** to act as a host for other guests. Samples of **1-MeOH** in microcrystalline form were soaked in one of a series of arenes and aliphatic molecules for 48 h. Most putative guests selected were hydrocarbons, but a fluorinated example of each was also tested, given the nature of the channel walls in **1**, which contain alternating hydrocarbon and fluorocarbon regions. Following exposure to each guest, the guest content of the sample was determined by dissolution of the sample in deuterated DMSO and examination by ^1^H NMR spectroscopy and GC. In each case, guest content was determined by the peak integration relative to the amount of TMP ligand. Selected examples were also studied as solids by ^13^C CP-MAS NMR spectroscopy to identify the guest and/or studied by TGA to determine guest content by mass loss. The results are summarized in table 2. Experimental data are provided in the electronic supplementary material.

**Table 2.** Guest contents determined by ^1^H NMR spectroscopy and GC.

	guest/TMP ratio				
guest	^1^H NMR (methyl)	^1^H NMR (aryl)	^13^C CP-MAS NMR	GC	TGA
toluene	0.68	0.65	guest observed	1.60	0.40
*o*-xylene	0.44	0.42	^a^	1.97	0.46
*m*-xylene	0.16	0.15	^a^	0.85	0.46
*p*-xylene	0.22	0.21	guest observed	0.38	0.24
tetramethylbenzene	0.16	0.12	guest observed	^a^	^a^
tetrafluorobenzene	0.24	n.a.	guest observed	0.32	^a^
pentane	^a^	^a^	^a^	0.00	^a^
cyclohexane	0	0	^a^	0.01	^a^
perfluoro(methylcyclohexane)	0	0	^a^	0.00	^a^

^a^No measurement made.

Although the measurements are not in quantitative agreement, notably with values for guest content determined by GC being higher than those determined by NMR and TGA, there are clear qualitative trends in the results that suggest that inclusion of arene guests is favourable, but that aliphatic guests are not included in the channels, and that fluorination does not have a pronounced effect on this trend.

### (c) Desolvation: open channels and channel collapse

**Figure 7.**
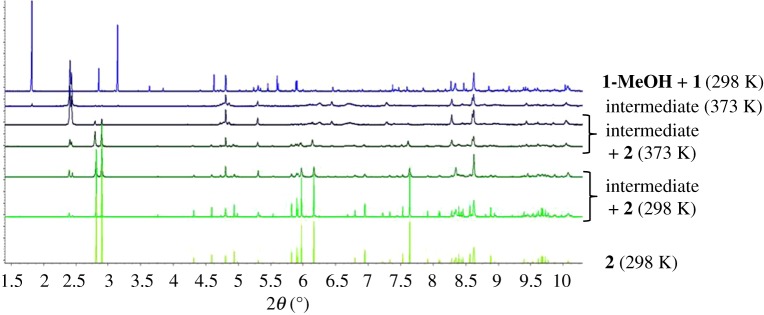
Sequence of powder patterns showing the reaction progression from **1-MeOH** to **2** via **1** and an intermediate. Note: a small amount (few %) of residual Ag_2_CO_3_ starting material is present throughout the sequence of patterns. (Online version in colour.)

The desolvation of **1-MeOH** was investigated *in situ* by PXRD at beamline ID31 (now ID22) at ESRF (figure 7). The sample was loaded so as to minimize MeOH loss prior to the onset of measurements. Pawley fitting of the initial patterns recorded at 298 K established that the sample used was a mixture of **1-MeOH** and **1**, which is unsurprising given the ease of MeOH loss, but also contained a very small amount of residual starting Ag_2_CO_3_ material (figure 2*a*). Heating of the sample capillary in a nitrogen stream at 323 K over a period of 18 min resulted in no change in the powder pattern indicative of a significant change in structure, although some change in the relative proportions of **1-MeOH** and **1** may occur. Heating at 373 K led to an immediate change in the pattern due to the formation of a new crystalline material as the predominant phase. The first pattern obtained at 373 K could be indexed. The unit cell of the new (intermediate) phase was established by Pawley refinement (*a* = 11.597(1), *b* = 19.080(2), *c* = 7.815(1) Å, *β* = 110.39(1), *V* = 1620.9(4); space gp. *C*2/*c*), but attempts at structure determination were unsuccessful. Further patterns, measured at 3 min intervals, indicated diminishing peak intensities for the new phase and growth of peaks corresponding to **2**. Upon cooling to 298 K, the peaks for the intermediate phase became absent, leaving compound **2** as a single phase.

## 4. Discussion

We have previously established a class of crystalline materials based on Ag(I) centres linked via perfluoroalkylcarboxylates and diimine linkers that exhibit flexibility in coordination number and geometry at the Ag(I) centres and/or in the fluoralkyl groups, each enabling these materials to undergo uptake and release of small molecule guests and changes in structure and/or composition that involve coordination bond formation or breaking [16–22]. The materials we have studied to date are not conventionally porous, but rely on the aforementioned mechanisms for flexibility in the crystalline solid state to enable latent porosity to be exploited. The current report exploits the coordination flexibility to establish the first conventionally porous material of this type, the MOF [Ag_2_(O_2_CCF_2_CF_2_CO_2_)(TMP)] **1**, prepared with MeOH in its channels as **1-MeOH**. The MOF structurally resembles other well-established one-dimensional-channel MOFs [37,38], which have been investigated for gas adsorption/separation and catalysis, but differs in the use of Ag(I) metal centres and having pore walls that comprise alkyl and fluoroalkyl regions.

**Figure 8.**
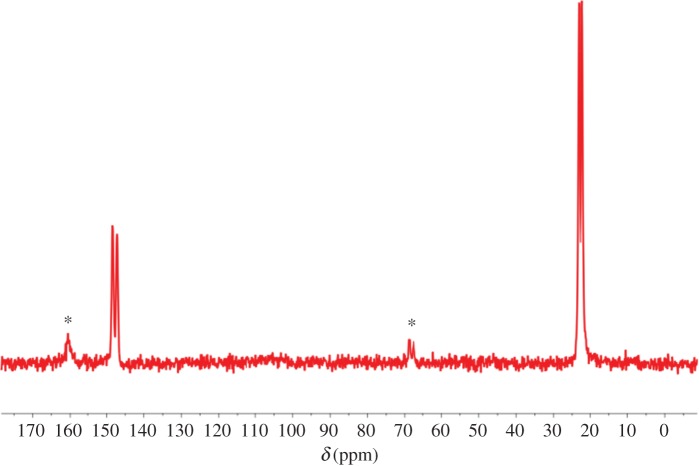
^13^C CP-MAS NMR spectrum of **2**, showing signals for TMP ligands. Signals for tetrafluorosuccinate ligands are not observed due to absence of nearby hydrogen nuclei. Asterisks indicate spinning sidebands. (Online version in colour.)

Single-crystal X-ray diffraction has established that **1-MeOH** can lose MeOH at room temperature to form the empty channel MOF **1**, but that **1** is unstable relative to rearrangement into the pillared material **2**, which is a non-porous higher-density polymorph of **1**. This rearrangement occurs in microcrystalline material but not with retention of the single-crystal form. Attempts to characterize **1-MeOH** or **1** by ^13^C CP-MAS NMR spectroscopy were unsuccessful due to the rapid loss of the solvent and subsequent conversion of **1** to **2**, which exhibits two ^13^C signals for methyl and ring carbons of the TMP ligands consistent with the asymmetric unit established by crystallography (figure 8).

**Figure 9.**
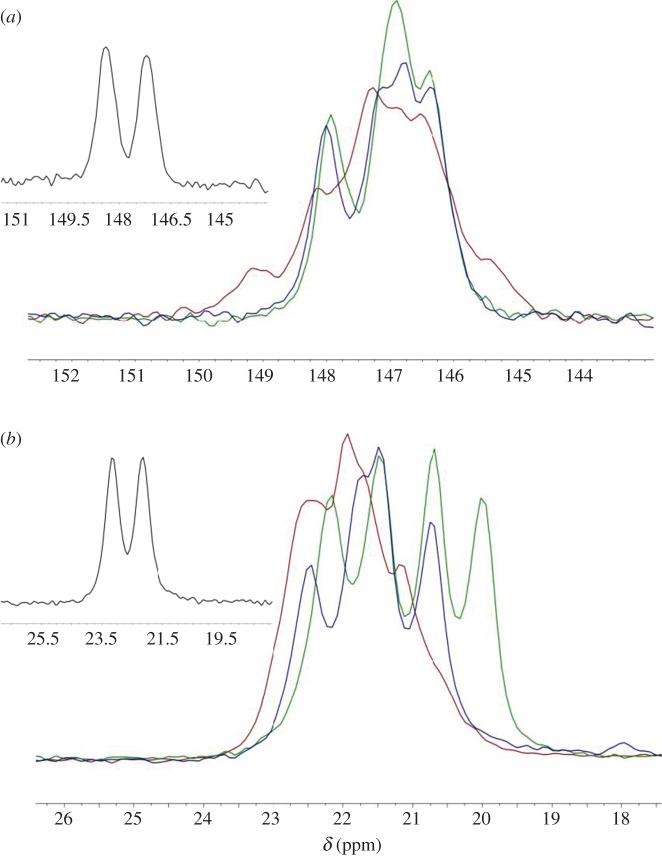
^13^ C CP-MAS NMR spectra showing signals for (*a*) TMP aryl carbons and (*b*) TMP methyl carbons in **1-guest** after exchange with different aromatic guests: ortho-xylene (red), toluene (blue) and tetrafluorobenzene (green). Insets show spectra of **2** for comparison.

The retention of the framework **1** upon exchange of MeOH for other solvents is confirmed by ^13^C solid-state NMR spectra, implying that these less volatile guests impart some stability on the open-channel form of the MOF. The chemical shifts of the TMP ligands are similar to those observed in **2** (figure 9; electronic supplementary material, figures S10–S13), but indicate more than two independent environments for both methyl and ring carbons, consistent with the crystal structure determinations of **1** and **1-MeOH**. Analysis of the guest content of guest-exchange MOFs [Ag_2_(O_2_CCF_2_CF_2_CO_2_)(TMP)]·*x*(guest) **1-guest** by a combination of techniques (TGA, ^1^H NMR and GC) clearly indicated that small arene guests (toluene, xylenes and tetrafluorobenzene) are adsorbed by the MOF and must reside in the channels, whereas aliphatic guests of similar size (pentane, cyclohexane, perfluoro(methylcyclohexane)) are not adsorbed. The mechanism for adsorption and the interaction of the guests with the framework is of interest, given the nature of the channel walls, which present a rugose surface of alternating C–H and C–F groups, and therefore an alternating positive and negative surface electrostatic potential, the C–H (methyl) groups projecting more prominently into the channels. The location of the arene guests could not be established crystallographically, but ^13^C solid-state NMR measurements indicate small changes in the chemical shifts of the aromatic ring and methyl group carbons of the TMP ligands, which correlate with how electron-rich or electron-poor the arene guest is (figure 9). The shift is of greater magnitude for the methyl carbons, which at the surface of the pores may be anticipated to be more affected by the guest content of the pores. The chemical shifts for **2** lie slightly downfield of those for the guest-containing MOF **1-guest**, for which the smallest upfield shift corresponds to the presence of the most electron-rich guest, xylene, and the largest upfield shift corresponds to the presence of the most electron-poor guest, tetrafluorobenzene.

The *in situ* PXRD study of heating **1-MeOH** revealed that, after removal of MeOH, the empty-pore MOF **1** exhibits stability at least for a period of minutes. Further heating leads to conversion to the pillared material **2,** which is a condensed non-porous polymorph of **1**. Intriguingly, the transformation from **1** to **2** occurs via an intermediate crystalline form, which forms rapidly on heating **1** at 373 K in a capillary, and converts more slowly, but still within a few minutes, to **2**. The diffraction peaks for the intermediate could be successfully indexed and its unit cell dimensions and space group determined, although crystal structure determination was unsuccessful. The composition of the intermediate must be the same as that of **1** and **2**, i.e. it is a third polymorph of [Ag_2_(O_2_CCF_2_CF_2_CO_2_)(TMP)]. It is important to note that no loss of TMP ligands was detected, which may suggest that there is no facile pathway for escape of the ligand. This contrasts with our observations for monocarboxylate-containing material [Ag_4_(O_2_CCF_2_CF_2_CF_3_)_4_(TMP)_3_], which upon heating loses TMP and rearranges to give [Ag_4_(O_2_CCF_2_CF_2_CF_3_)_4_(TMP)_2_] [19,20]. Structure determination of the intermediate material would shed light on the mechanism of the solid-state rearrangement, which requires considerable reorganization of metal–ligand bonding, and again highlights the flexibility and propensity for breaking and formation of coordination bonds in the crystalline solid state.

## 5. Conclusion

We report the synthesis and characterization of a new MOF [Ag_2_(O_2_CCF_2_CF_2_CO_2_)(TMP)] **1** containing one-dimensional channels of diameter in the range 7–10 Å which present an alternating surface of alkyl and fluoralkyl groups. The MOF has been prepared with MeOH in the channels (**1-MeOH**) and is readily desolvated, as demonstrated by single-crystal X-ray diffraction and PXRD as well as ^13^C CP-MAS NMR spectroscopy. The empty-channel MOF exhibits quite limited stability under ambient conditions or under heating but is stable for longer periods at low temperature under an N_2_ atmosphere, enabling single-crystal structure determination. Crystalline **1-MeOH**, when exposed to other liquid guests of suitable size to enter the channels, takes up arene guests, but does not take up aliphatic guests of similar size. Use of fluorinated arenes or aliphatic molecules did not change these outcomes. Heating of crystalline **1-MeOH** leads, after solvent loss, to conversion of empty-pore MOF **1** into a pillared non-porous polymorph **2**, a transformation that takes place via a clearly identified crystalline intermediate, for which the unit cell and space group but not crystal structure could be determined from an *in situ* powder diffraction study.

There is much current interest in MOFs that exhibit flexibility and have the potential to behave as guest-responsive materials [12–15]. This study adds to our understanding in MOFs and coordination polymers of dynamic behaviour and structural transformations resulting from rearrangements of metal–ligand bonding [39–41].

## Supplementary Material

Ag-channel-MOF_30Oct16_SI.pdf

## Supplementary Material

1-MeOH.cif

## Supplementary Material

1.cif

## Supplementary Material

2.cif

## Footnotes

1 SADABS Sheldrick GM. SADABS empirical absorption correction program [28], University of Göttingen, based on the method of Blessing [29].

## Data accessibility

The electronic supplementary material contains figures showing the crystal structure of **2**, TGA data, ^1^H NMR and ^13^C CP-MAS NMR data and GC data. Cambridge Crystallographic Data Centre reference nos. CCDC-1499745–1499747 contain the single-crystal structure data for this paper. These data can be obtained free of charge via www.ccdc.cam.ac.uk/conts/retrieving.html (or from the Cambridge Crystallographic Data Centre, 12 Union Road, Cambridge CB21EZ, UK; fax: (+44)1223-336-033; or deposit@ccdc.cam.ac.uk).
