# Supplementary material for: Arene guest selectivity and pore flexibility in a metal–organic framework with semi-fluorinated channel walls
Source: Philos Trans A Math Phys Eng Sci. 2017 Jan 13;375(2084):20160031. doi: 10.1098/rsta.2016.0031 (PMC5179934; doi:10.1098/rsta.2016.0031)
Supplement: Ag-channel-MOF_30Oct16_SI.pdf [file rsta20160031supp1.pdf]

Supporting Information

Guest-exchange and pore flexibility in a MOF with semi-fluorinated channel walls

Rebecca Smith,[a] Iñigo J. Vitórica-Yrezábal,[a,b] Adrian Hill[c] and Lee Brammer[a,*]

[a] *Department of Chemistry, University of Sheffield, Brook Hill, Sheffield S3 7HF, UK.*

[b] *Current address: School of Chemistry, University of Manchester, Oxford Road, Manchester M13 9PL, UK.*

[c] *Johnson Matthey Process Technologies, Inc., Savannah, GA 31408, USA*

Table of Contents

1. Crystal structure of **1** page S2
2. Guest exchange page S2
3. TGA Data page S3
4. ^{13}C NMR Spectroscopic Data (solid state) page S8
5. ^{1}H NMR Spectroscopic Data (solution phase) page S11
6. GC data page S13

1. Crystal Structure of 1

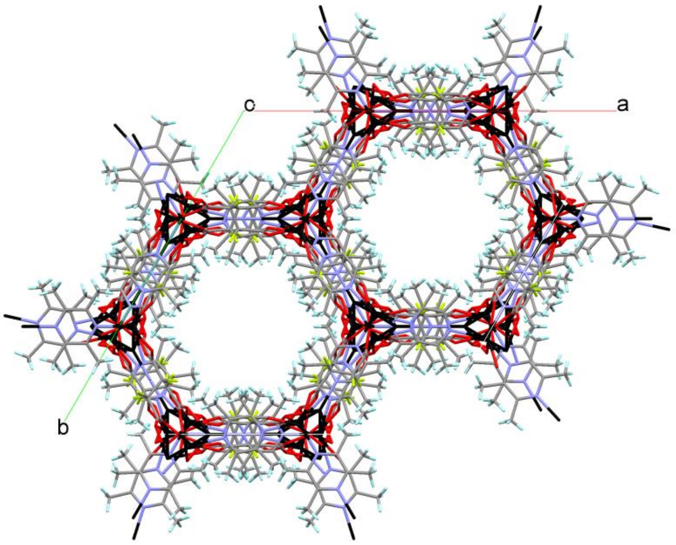

Figure S1. (a) Crystal structure of **1** viewed down *c*-axis showing hexagonal channels. Silver atoms in black, carbon in grey, hydrogen in cyan, nitrogen in blue, fluorine in yellow.

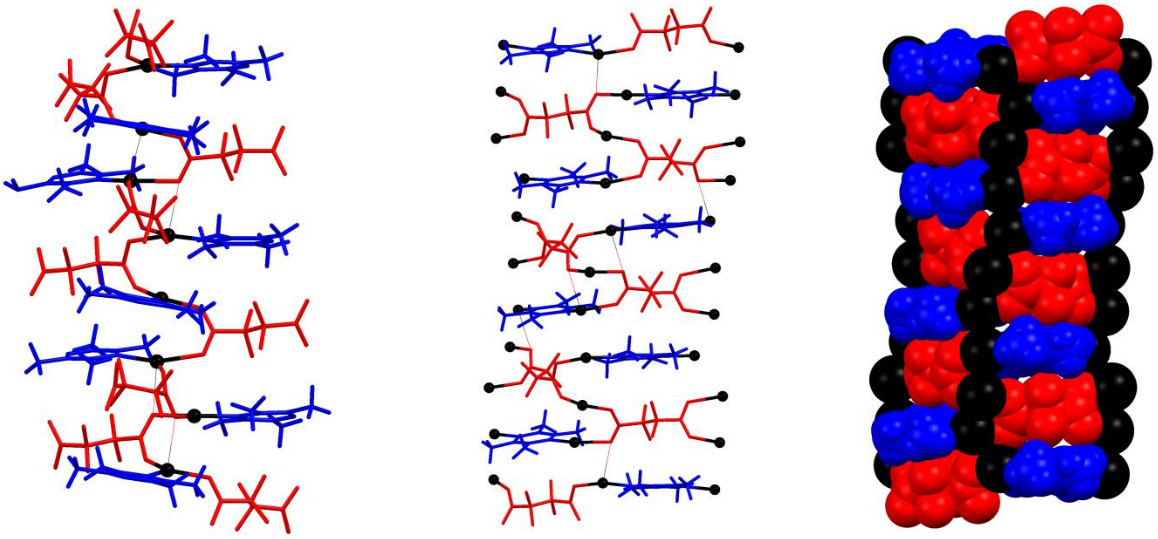

Figure S2. (a) A section of the column of Ag(I) centres that lies along the intersection of channel walls (along *c*-axis) in **1** showing the trigonal (and occasional pseudotetrahedral) coordination of Ag(I) centres. (b) Section showing two walls of a channel emphasising alternating arrangement of ligands. (c) Same section as shown in (b), but in spacefilling representation to illustrate rugose channel wall surfaces. Long Ag–O bonds (2.696 Å) are shown thin lines. Ag atoms in black, tetrafluorosuccinate ligands in red, TMP ligands in blue.

2. Guest exchange

$[Ag_2(O_2CCF_2CF_2CO_2)(TMP)]\cdot(MeOH)_x(guest)_y$, (guest = toluene, o- xylene, m- xylene, p-xylene, 1,2,4,5- tetramethylbenzene, 1,2,4,5-tetrafluorobenzene, pentane, cyclohexane and perfluoro(methylcyclohexane))

Crystals of **1-MeOH** were prepared in methanol as described in the Experimental section. The methanol was then decanted and 10 mL of the required guest added (all guests are liquid at room

temperature except for tetramethylbenzene, for which a saturated solution of tetramethylbenzene in toluene was prepared instead). After 24 h the guest solutions were decanted again and replaced with another 10 mL of guest solution and left for a further 24 h. Samples were then analysed by TGA and ^{13}C CP-MAS solid-state NMR spectroscopy. After digestion of the MOF in DMSO, analysis of guest content by solution-phase ^{1}H NMR spectroscopy and gas chromatography was conducted.

3. Thermogravimetric Analysis Data

Thermogravimetric analyses were conducted using a Perkin-Elmer Pyris1 TGA model thermogravimetric analyser. Samples were heated from 25 to 450 ˚C at 5 ˚C min^{-1}, under a nitrogen atmosphere.

$[Ag_2(O_2CCF_2CF_2CO_2)(TMP)]$ (1)

Thermogravimetric analysis was conducted on a dry sample of **1** to determine the stability of the framework and how it degrades upon increasing the temperature to 450 °C. There is a mass loss of approximately 24.9% with an onset temperature of around 160 °C which corresponds to the loss of tmp (expected 25.2%) leaving $[Ag_2\{O_2C(CF_2)_2CO_2\}]$. From 330 °C there is another mass loss of 34.6% corresponding to the loss of tetrafluorosuccinate (expected 35.19%) to leave Ag_2O.

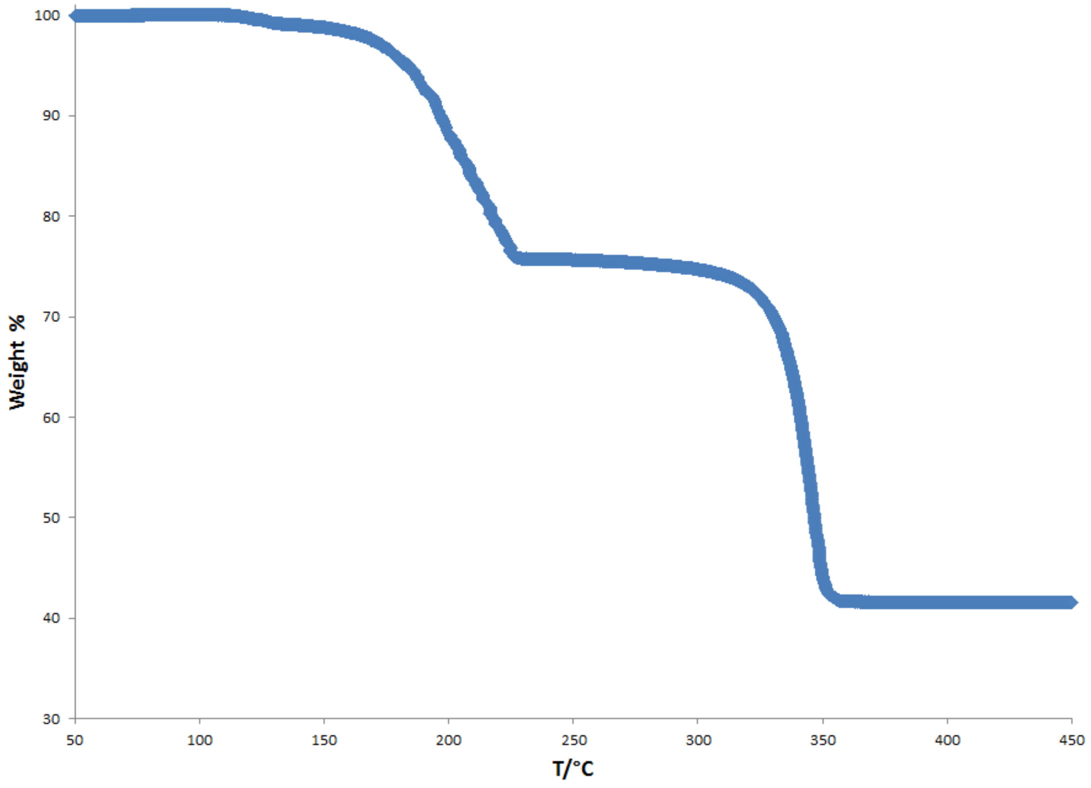

Figure S3. TGA trace for $[Ag_2(O_2CCF_2CF_2CO_2)(TMP)]$

$[Ag_2(O_2CCF_2CF_2CO_2)(TMP)]\cdot(toluene)_x$

A sample of $[Ag_2(O_2CCF_2CF_2CO_2)(TMP)]\cdot(toluene)_x$ was heated to 300 ˚C to determine the relative proportion of toluene and TMP in the material. There is a mass loss of around 7.4% between 110 °C-140 °C assigned to toluene and a further loss of 22.6 % in the temperature range 160–240 °C caused by TMP (expected 23.4%) leaving $[Ag_2\{O_2C(CF_2)_2CO_2\}]$. This suggests there are approximately 0.4 toluene molecules per formula unit.

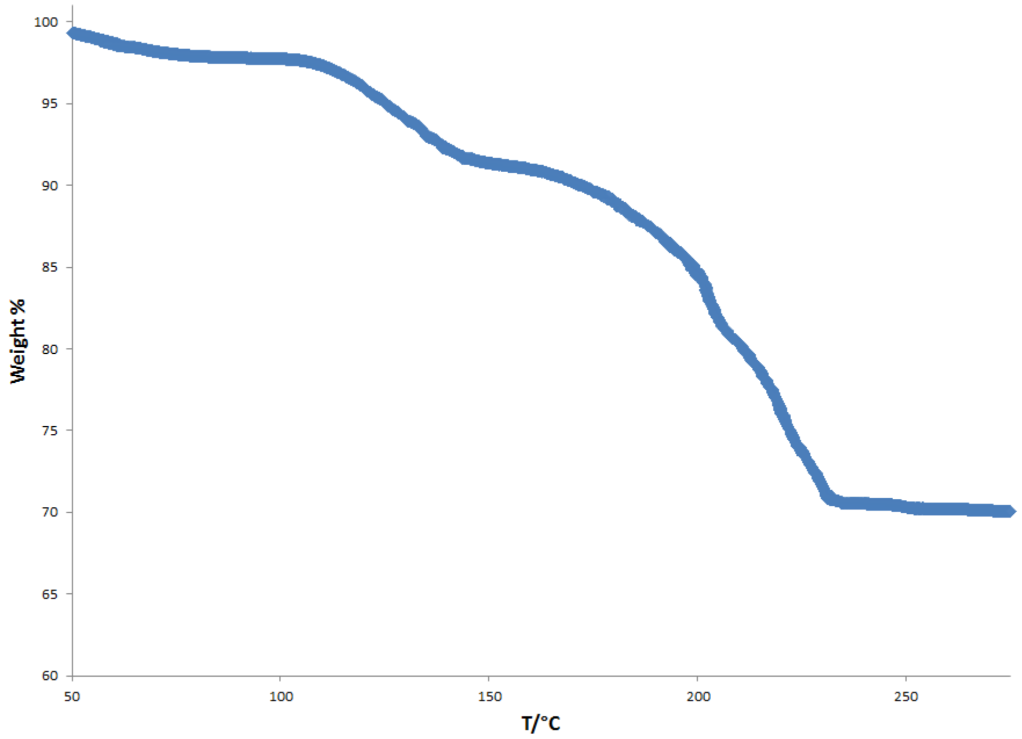

Figure S4. TGA trace for $[Ag_2(O_2CCF_2CF_2CO_2)(TMP)]\cdot(toluene)_x$

$[Ag_2(O_2CCF_2CF_2CO_2)(TMP)]\cdot($*m*-xylene$)_x$

A sample of $[Ag_2(O_2CCF_2CF_2CO_2)(TMP)]\cdot$(*m*-xylene)$_x$ was heated to 300 ˚C to determine the relative proportion of m-xylene and tmp in the material.There is a mass loss of around 8.3% in the temperature range 110–140 °C assigned to *m*-xylene and a further loss of 23.6 % in the temperature range 150–240 °C caused by TMP (expected 22.9 %) leaving $[Ag_2\{O_2C(CF_2)_2CO_2\}]$. This suggests there are approximately 0.46 *m*-xylene molecules per formula unit.

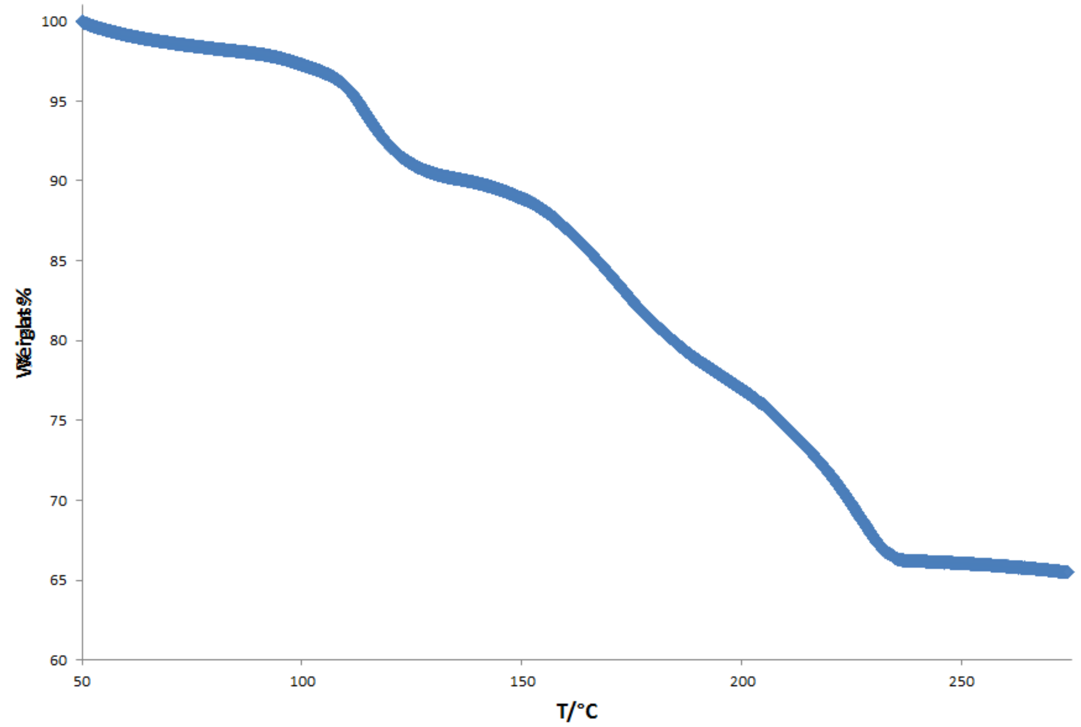

Figure S5. TGA trace for $[Ag_2(O_2CCF_2CF_2CO_2)(TMP)]\cdot(\textit{m}\text{-xylene})_x$

$[Ag_2(O_2CCF_2CF_2CO_2)(TMP)]\cdot(\textit{p}\text{-xylene})_x$

A sample of $[Ag_2(O_2CCF_2CF_2CO_2)(TMP)]\cdot(\textit{p}\text{-xylene})_x$ was heated to 300 ˚C to determine the relative proportion of m-xylene and tmp in the material.There is a mass loss of around 4.6 % in the temperature range 120–160 °C assigned to *p*-xylene and a further loss of 23.0 % in the temperature range 160–240 °C caused by TMP (expected 23.1 %) leaving $[Ag_2\{O_2C(CF_2)_2CO_2\}]$. This suggests there are approximately 0.24 *p*-xylene molecules per formula unit.

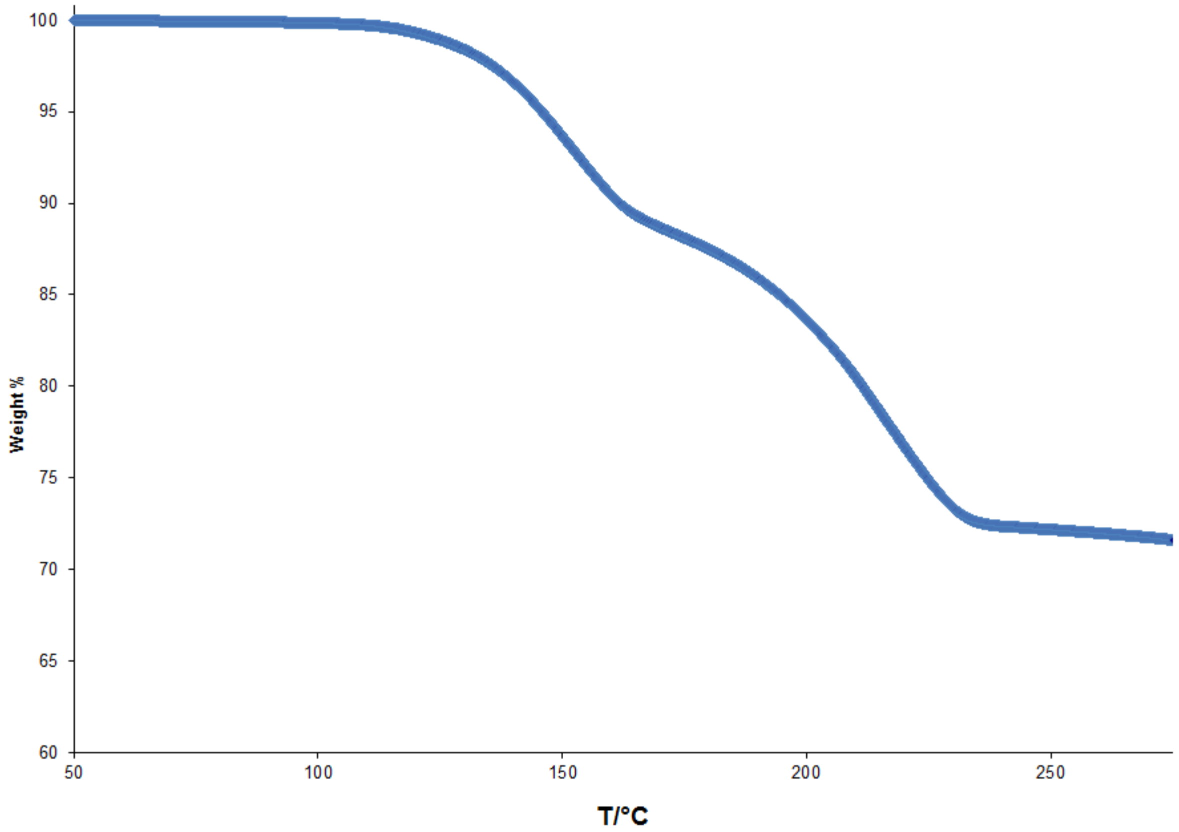

Figure S6. TGA trace for [$Ag_2(O_2CCF_2CF_2CO_2)$(TMP)]·(*p*-xylene)$_x$

[$Ag_2(O_2CCF_2CF_2CO_2)$(TMP)]·(*o*-xylene)$_x$

A sample of [$Ag_2(O_2CCF_2CF_2CO_2)$(TMP)]·(*o*-xylene)$_x$ was heated to 300 ˚C to determine the relative proportion of *o*-xylene and TMP in the material. There is a mass loss of around 8.3 % in the temperature range 110–140 °C assigned to *o*-xylene and a further loss of 22.5 % in the temperature range 160–240 °C caused by TMP (expected 22.2 %) leaving [$Ag_2\{O_2C(CF_2)_2CO_2\}$]. This suggests there are approximately 0.46 *o*-xylene molecules per formula unit.

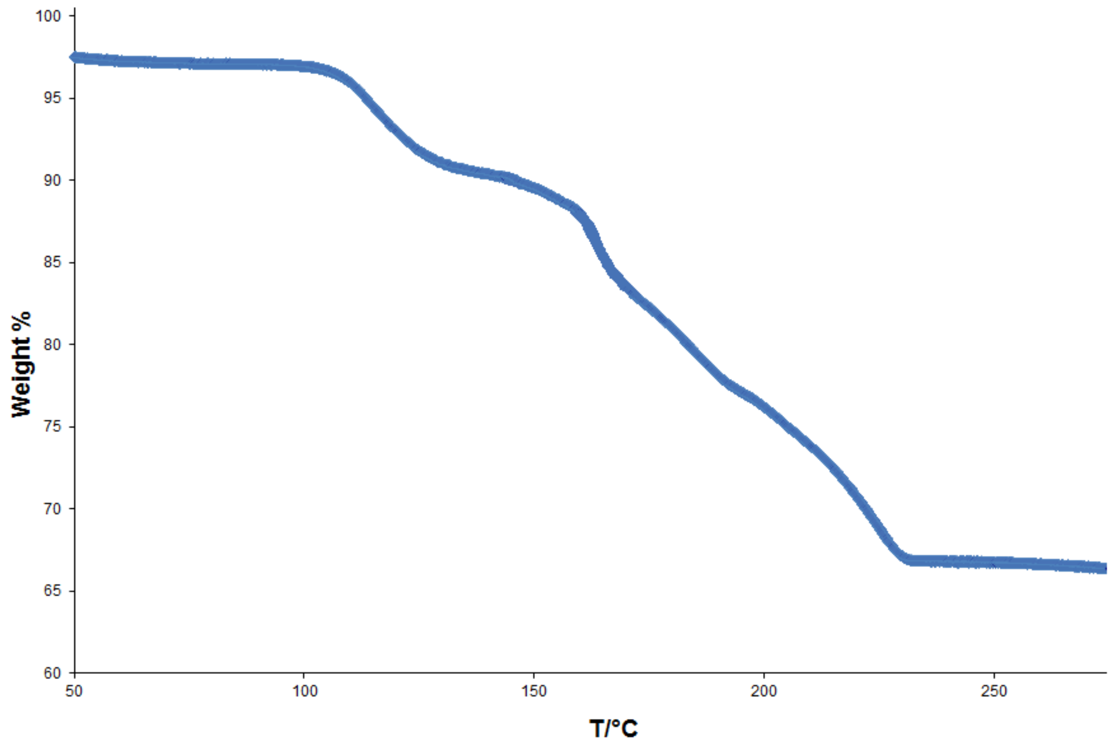

Figure S7. TGA trace for $[Ag_2(O_2CCF_2CF_2CO_2)(TMP)]\cdot(o\text{-xylene})_x$

4. ^{13}C solid-state NMR Spectroscopic Data

^{13}C NMR spectra were recorded on a Bruker 500 MHz FT-NMR spectrometer (125.669 MHz for ^{13}C). All experiments used cross-polarisation from ^{1}H and magic angle spinning at 8–10 Hz. Data were processed using Bruker TOPSPIN 1.3 and MestRe Nova. All chemical shifts were referenced to a solid sample of adamantane.

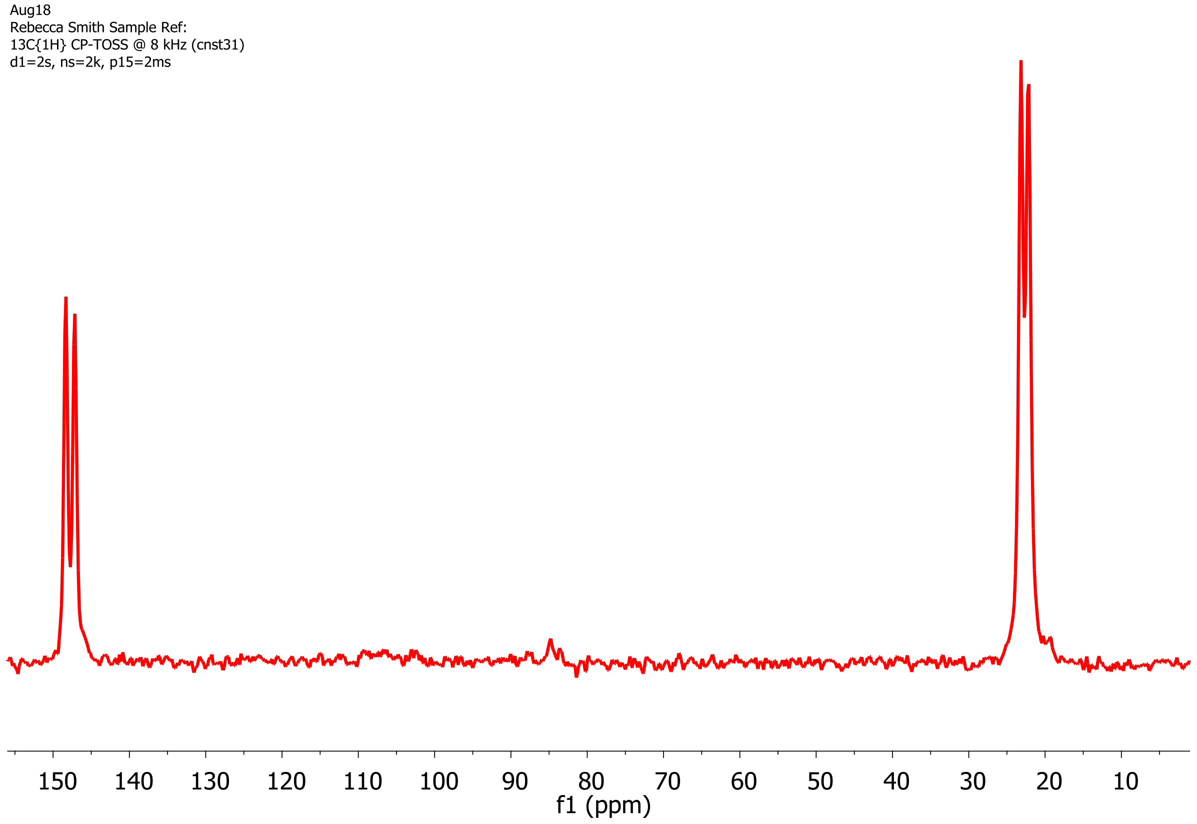

Figure S8. Solid-state ^{13}C-NMR spectrum for [$Ag_2(O_2CCF_2CF_2CO_2)$(TMP)] **2**. Total sideband suppression (CP-TOSS) experiment.

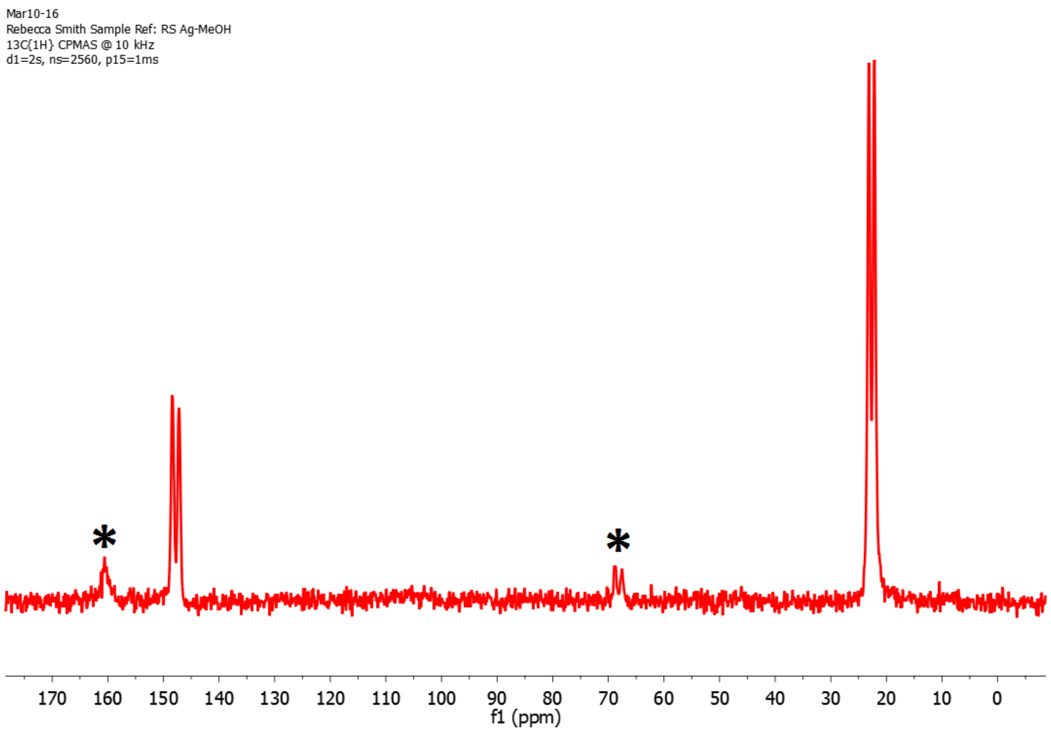

Figure S9. Attempt to collect solid-state ^{13}C-NMR spectrum for **1-MeOH** or **1**, avoiding significant MeOH loss. Spectrum indicates MeOH loss has led to collapse of **1** to give **2**. Asterisks indicate spinning side bands.

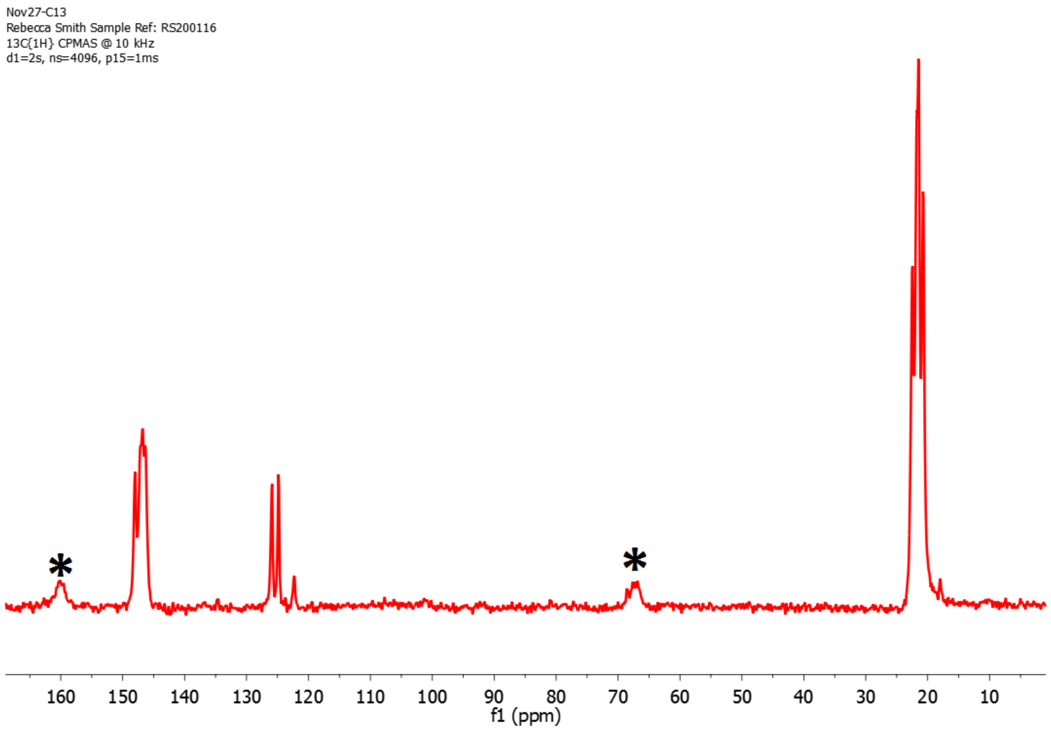

Figure S10. Solid-state ^{13}C-NMR spectrum for **[$Ag_2(O_2CCF_2CF_2CO_2)$(TMP)]·(toluene)$_x$**. Asterisks indicate spinning side bands.

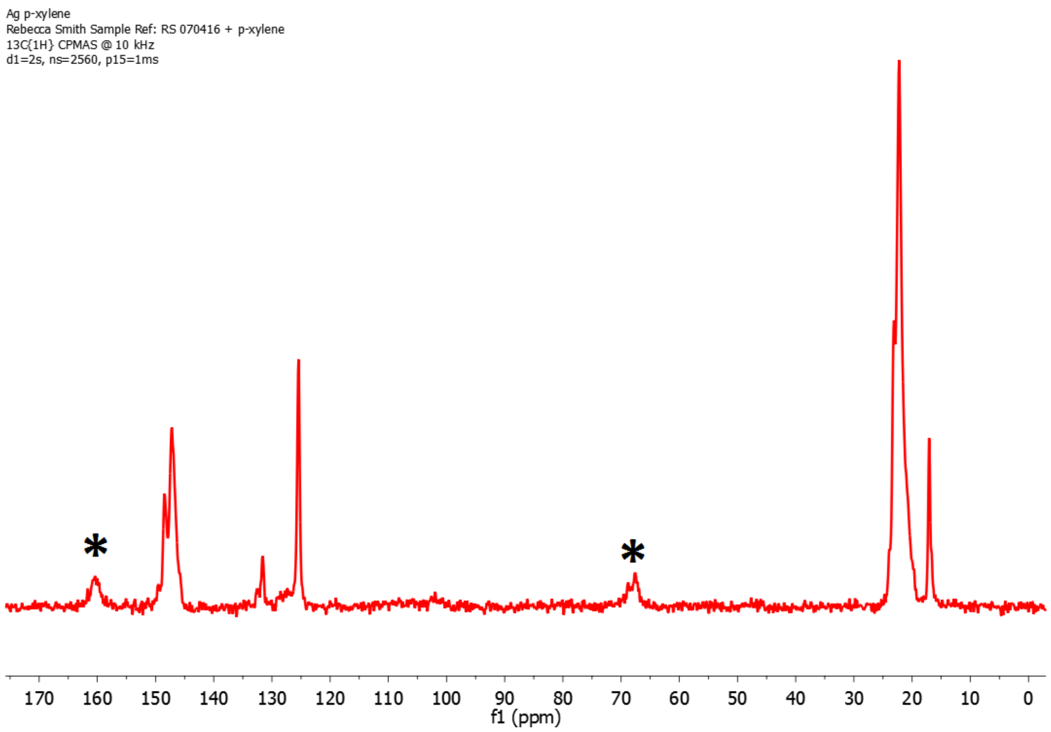

Figure S11. Solid-state ^{13}C-NMR spectrum for **[$Ag_2(O_2CCF_2CF_2CO_2)$(TMP)]· (*p*-xylene)$_x$**. Asterisks indicate spinning side bands.

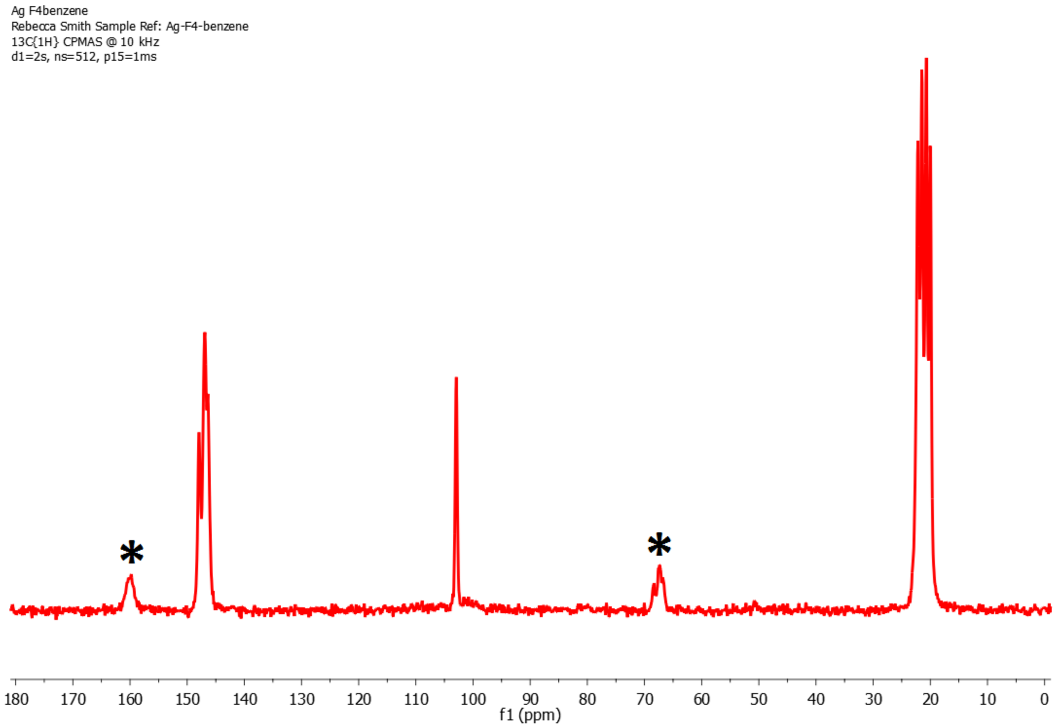

Figure S12. Solid-state ^{13}C-NMR spectrum for **[$Ag_2(O_2CCF_2CF_2CO_2)$(TMP)]·(1,2,4,5-tetrafluorobenzene)$_x$**. Asterisks indicate spinning side bands.

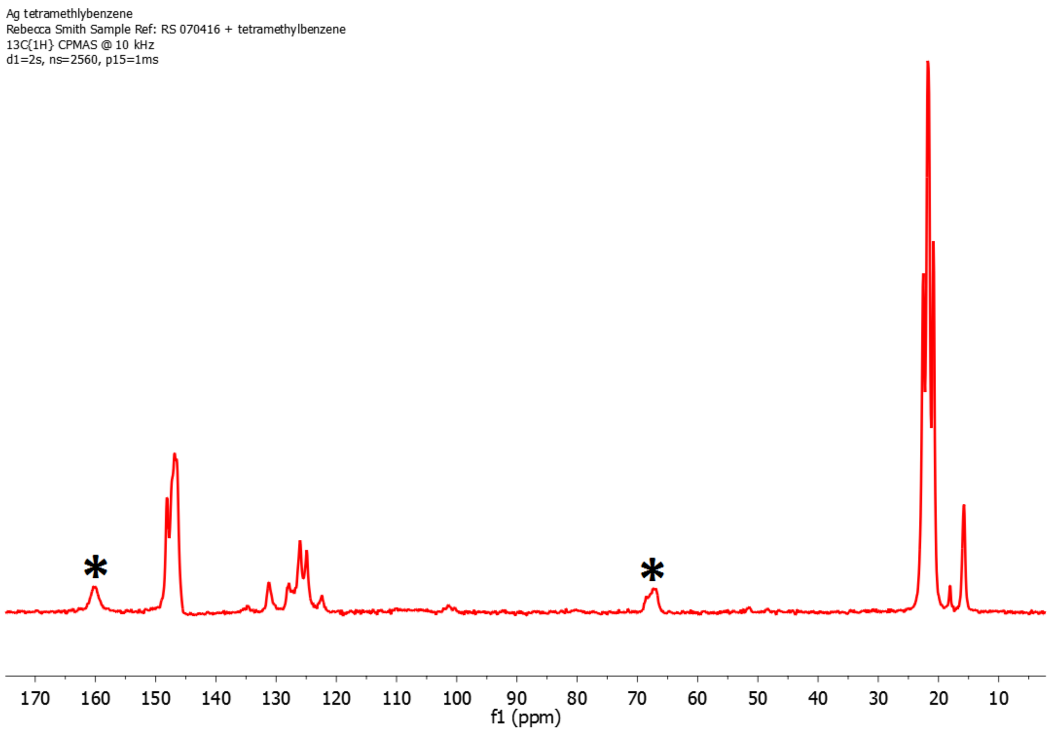

Figure S13. Solid-state ^{13}C-NMR spectrum for **[$Ag_2(O_2CCF_2CF_2CO_2)$(TMP)]·(1,2,4,5-tetramethylbenzene)$_x$**. Asterisks indicate spinning side bands.

5. ^{1}H NMR Spectroscopic Data

^{1}H spectra were recorded on a Bruker 400 MHz FT-NMR spectrometer and. Guest-exchanged samples (~2-5 mg) of **1** were dissolved in DMSO-d6 before analysis. Chemical shifts are reported in ppm relative to TMS (0 ppm) with the residual solvent resonances used as an internal reference The NMR data were analysed using the Bruker Topspin programme suite. Guest concentrations were determined by comparing integrations of the guest and TMP signals.

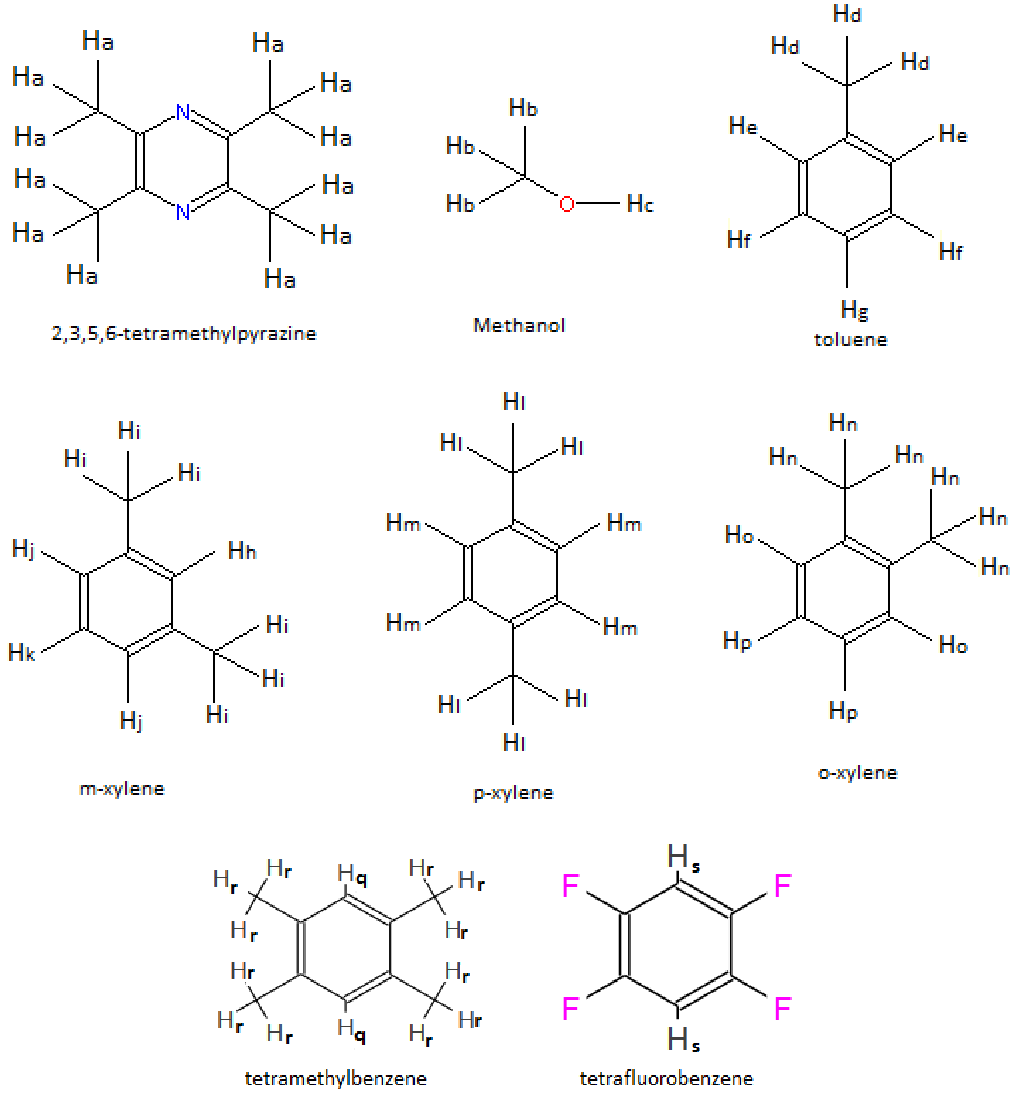

Figure S14. Proton environments for ^{1}H-NMR analytes in this study, H_a-H_p

Table S1. ^{1}H-NMR data for **[$Ag_2(O_2CCF_2CF_2CO_2)$(TMP)] (1)** after dissolution in DMSO

δ/ppm	Multiplicity	Assignment
2.38	singlet	H_a
2.51	singlet	DMSO
3.34	singlet	H_2O

Table S2. ^{1}H-NMR data for **[$Ag_2(O_2CCF_2CF_2CO_2)$(TMP)]·(toluene)$_x$** (x=0.68) after dissolution in DMSO.

δ/ppm	Multiplicity	Assignment	Integration
2.29	singlet	H_d	0.17

2.37	singlet	H_a	1
2.49	singlet	DMSO	-
3.36	singlet	H_2O	-
7.1-7.3	multiplet	H_e, H_f, H_g	0.27

Table S3. ^{1}H-NMR data for **$[Ag_2(O_2CCF_2CF_2CO_2)(TMP)]\cdot(m\text{-xylene})_x$** (x=0.16) after dissolution in DMSO.

δ/ppm	Multiplicity	Assignment	Integration
2.09	singlet	H_i	0.08
2.38	singlet	H_a	1
2.51	singlet	DMSO	-
3.35	singlet	H_2O	-
7.0-7.2	multiplet	H_h,H_j,H_k	0.05

Table S4. ^{1}H-NMR data for **$[Ag_2(O_2CCF_2CF_2CO_2)(TMP)]\cdot(p\text{-xylene})_x$** (x=0.22) after dissolution in DMSO.

δ/ppm	Multiplicity	Assignment	Integration
2.24	singlet	H_l	0.11
2.38	singlet	H_a	1
2.52	singlet	DMSO	-
3.34	singlet	H_2O	-
7.05	singlet	H_m	0.07

Table S5. ^{1}H-NMR data for **$[Ag_2(O_2CCF_2CF_2CO_2)(TMP)]\cdot(\text{o-xylene})_x$** (x=0.44) after dissolution in DMSO. This sample appears to contain some methanol, approximately 0.08 per formula unit.

δ/ppm	Multiplicity	Assignment	Integration
2.21	singlet	H_n	0.22
2.39	singlet	H_a	1
2.52	singlet	DMSO	-
3.15	quartet	H_c	0.01
3.37	singlet	H_2O	-
4.11	doublet	H_b	0.02
7-7.2	multiplet	H_o, H_p	0.14

Table S6. ^{1}H-NMR data for **$[Ag_2(O_2CCF_2CF_2CO_2)(TMP)]\cdot(\text{tetramethylbenzene})_x$** (x=0.16) after dissolution in DMSO

δ/ppm	Multiplicity	Assignment	Integration
2.21	singlet	H_r	0.16
2.38	singlet	H_a	1
2.51	singlet	DMSO	-
3.35	singlet	H_2O	-

7.03	singlet	H_q	0.02

Table S7. ^{1}H-NMR data for **[$Ag_2(O_2CCF_2CF_2CO_2)$(TMP)]·(cyclohexane)$_x$** (x=0) after dissolution in DMSO

δ/ppm	Multiplicity	Assignment
2.38	singlet	H_a
2.51	singlet	DMSO
3.37	singlet	H_2O

Table S8. ^{1}H-NMR data for **[$Ag_2(O_2CCF_2CF_2CO_2)$(TMP)]·(perfluoro(methylcyclohexane))$_x$** after dissolution in DMSO. Impurity could arise from perfluoro(methylcyclohexane), which has a purity of 90% for the commercial material used.

δ/ppm	Multiplicity	Assignment
2.39	singlet	H_a
2.52	singlet	DMSO
3.18	doublet	impurity
3.34	singlet	H_2O

Table S9. ^{1}H-NMR data for **[$Ag_2(O_2CCF_2CF_2CO_2)$(TMP)]·(tetrafluorobenzene)$_x$** (x=0.24) after dissolution in DMSO.

δ/ppm	Multiplicity	Assignment	Integration
2.31	singlet	H_a	1
2.50	singlet	DMSO	-
3.26	singlet	H_2O	-
7.64	singlet	H_s	0.04

6. Gas Chromatographic Data

Samples of guest-soaked MOF (approx. 2-5 mg) were lightly dried in air for 5 min before dissolving in DMSO. The solutions were transferred to screw cap glass vials, and then analysed using a Perkin-Elmer Autosystem GC with an AlltechTM HeliflexTM AT-1 capillary column (L x I.D. 30 m x 0.32 mm x df 5.00 μm), heating from 40 to 200 °C at 10 °C min^{-1}. Expected guest retention times were determined from DMSO solutions of each guest and found to be 2.2 min (methanol), 3.6 min (perfluoro(methylcyclohexane)), 9.6 min (1,2,4,5-tetrafluorobenzene),10.3 min (cyclohexane), 11.39 min (pentane), 12.8 min (toluene), 15.1 min (p-xylene), 15.2 min (m-xylene) and 15.6 min (o-xylene). Relative content of guests was determined by comparing peak areas to that of TMP (retention time 18.9 min).

Table S10. Summary of guest contents determined by GC

Guest	Guest/TMP ratio
toluene	1.60
o-xylene	1.97
m-xylene	0.85
p-xylene	0.38
tetrafluorobenzene	0.32
pentane	0.00
cyclohexane	0.01
perfluoro-(methylcyclohexane)	0.00

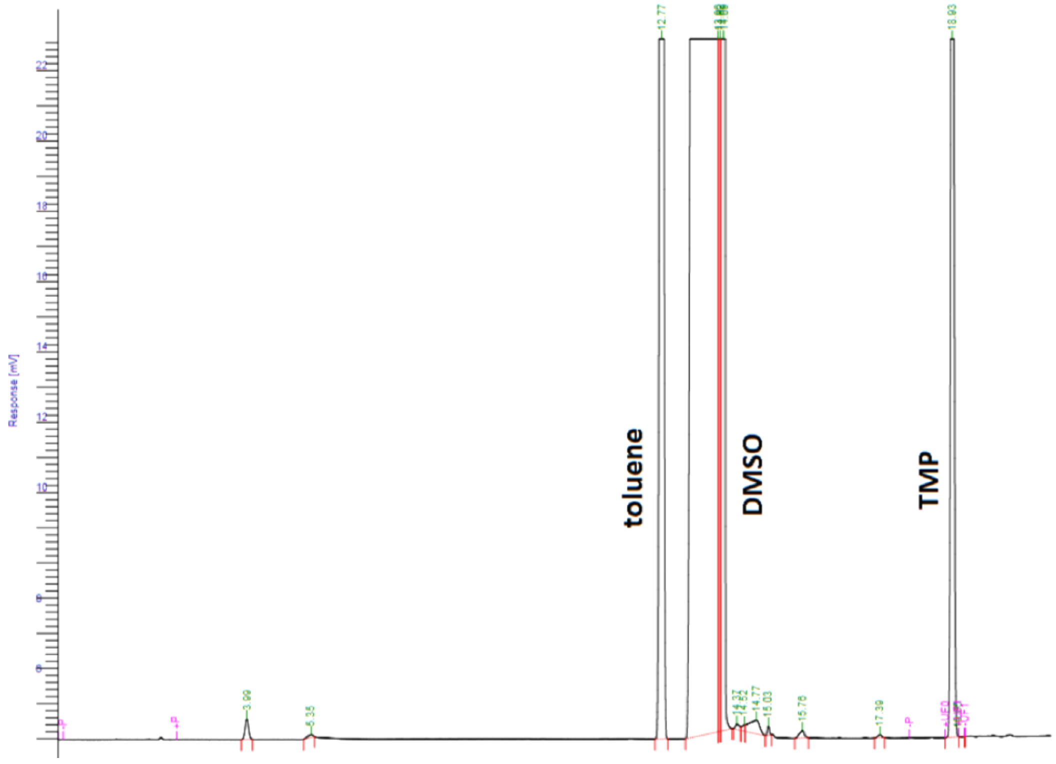

Figure S15. Gas chromatogram for **[$Ag_2(O_2CCF_2CF_2CO_2)$(TMP)]·(toluene)$_x$** after dissolution in DMSO.

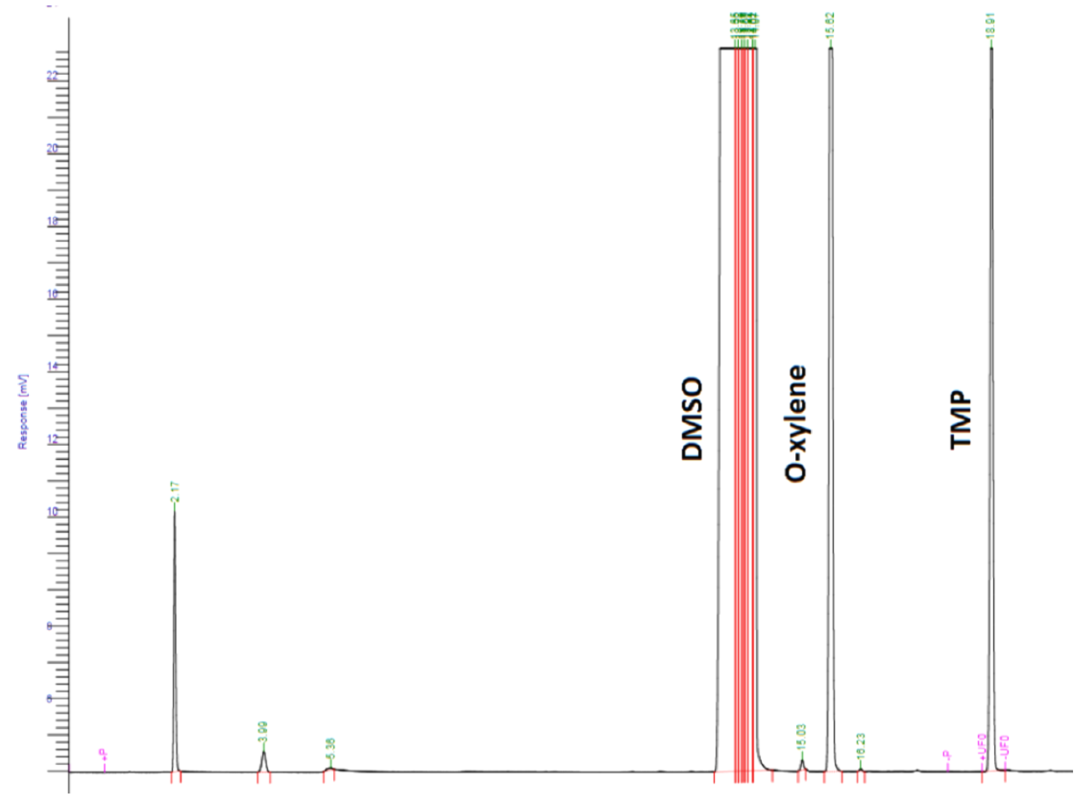

Figure S16. Gas chromatogram for **[Ag$_2$(O$_2$CCF$_2$CF$_2$CO$_2$)(TMP)]·(*o*-xylene)$_x$** after dissolution in DMSO.

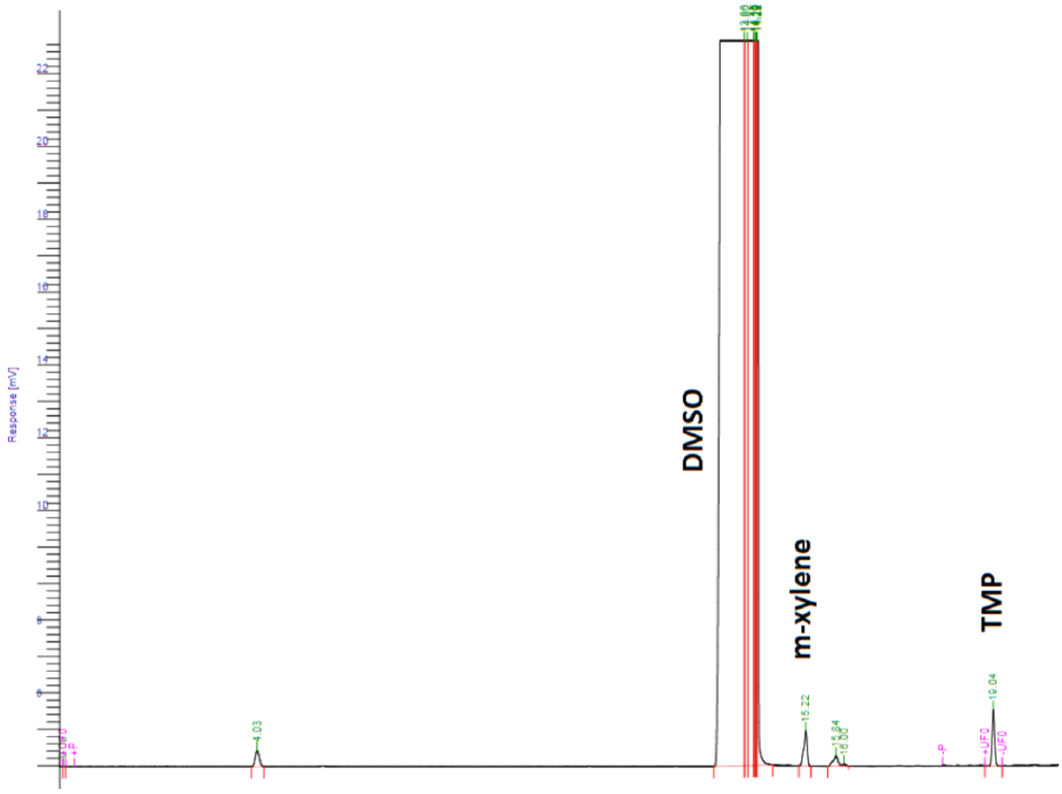

Figure S17. Gas chromatogram for **[$Ag_2(O_2CCF_2CF_2CO_2)$(TMP)]·(*m*-xylene)$_x$** after dissolution in DMSO.

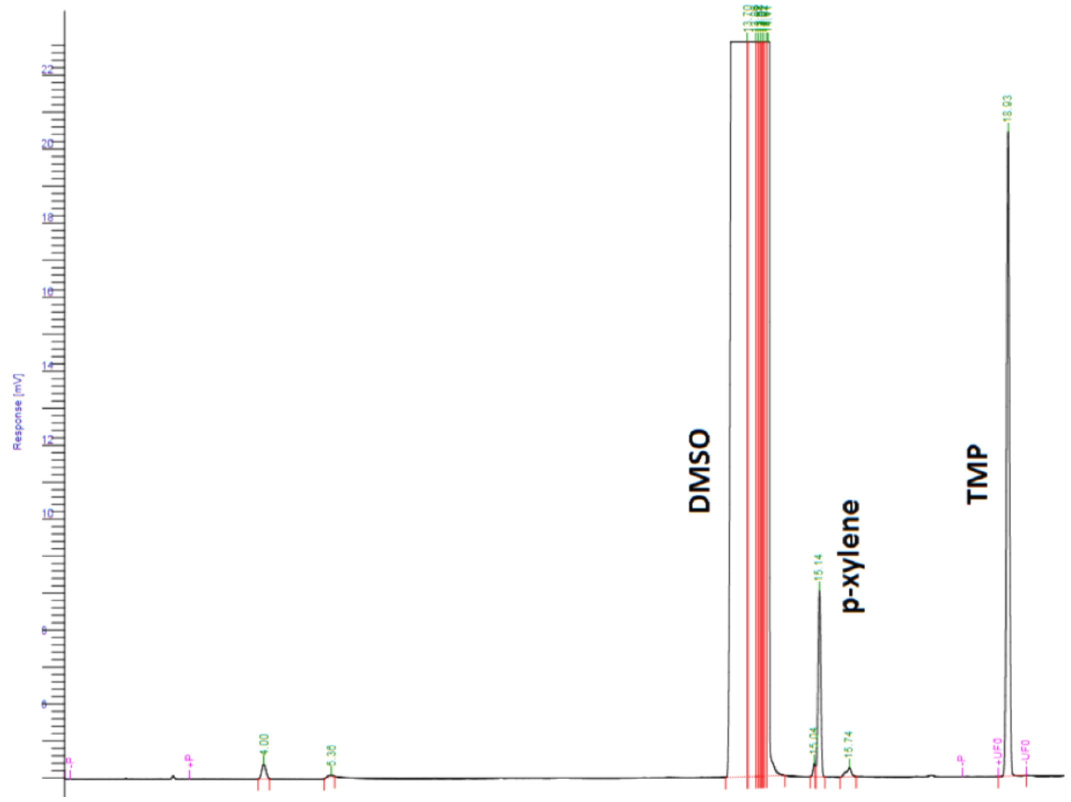

Figure S18. Gas chromatogram for $\mathbf{[Ag_2(O_2CCF_2CF_2CO_2)(TMP)]\cdot(\mathit{p}\text{-xylene})_x}$ after dissolution in DMSO.

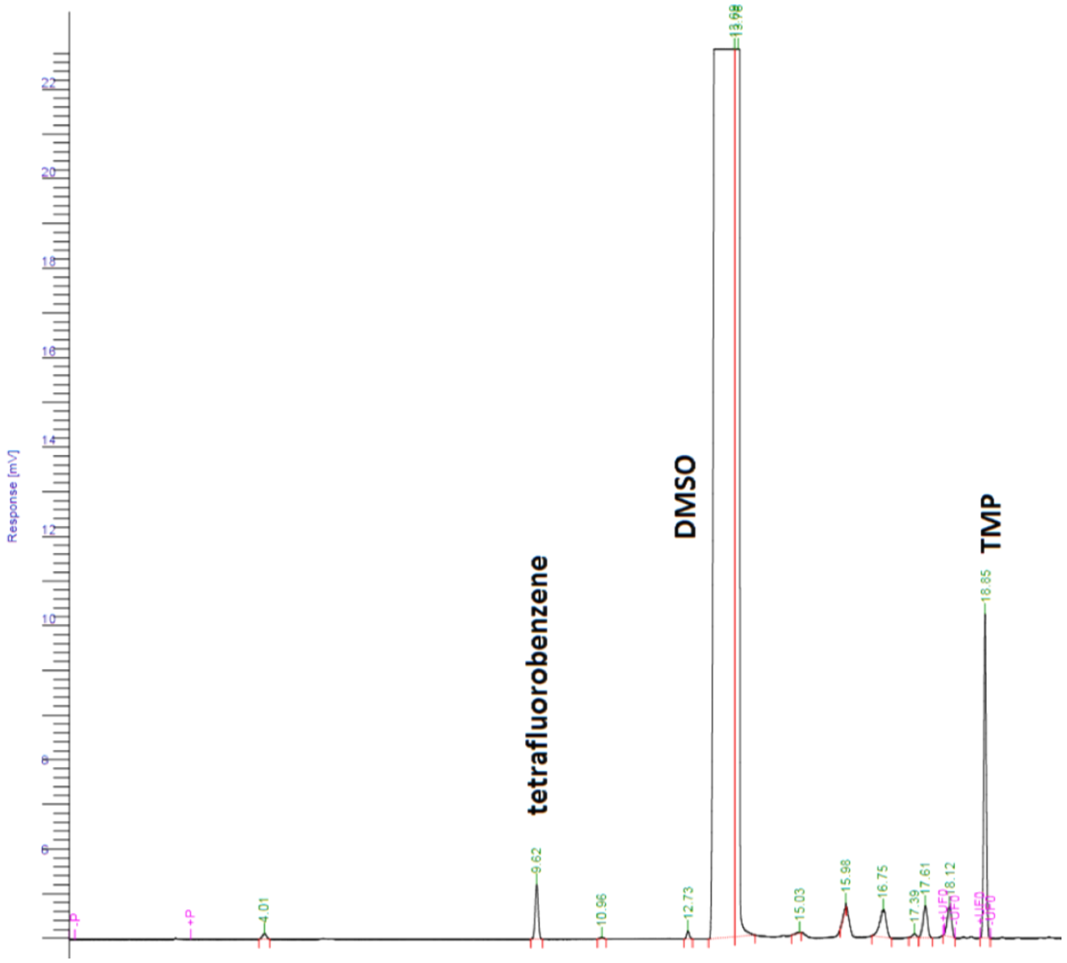

Figure S19. Gas chromatogram for **[$Ag_2(O_2CCF_2CF_2CO_2)$(TMP)]·(tetrafluorobenzene)$_x$** after dissolution in DMSO.

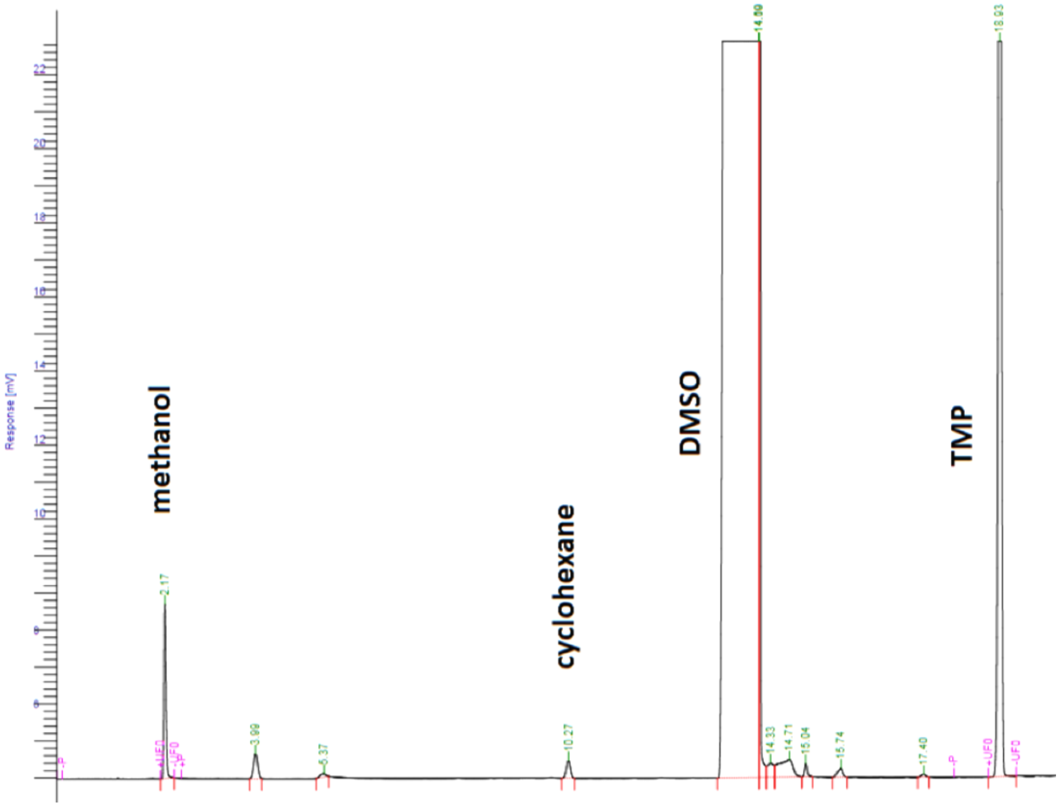

Figure S20. Gas chromatogram for **[$Ag_2(O_2CCF_2CF_2CO_2)$(TMP)]·(cyclohexane)$_x$** after dissolution in DMSO.

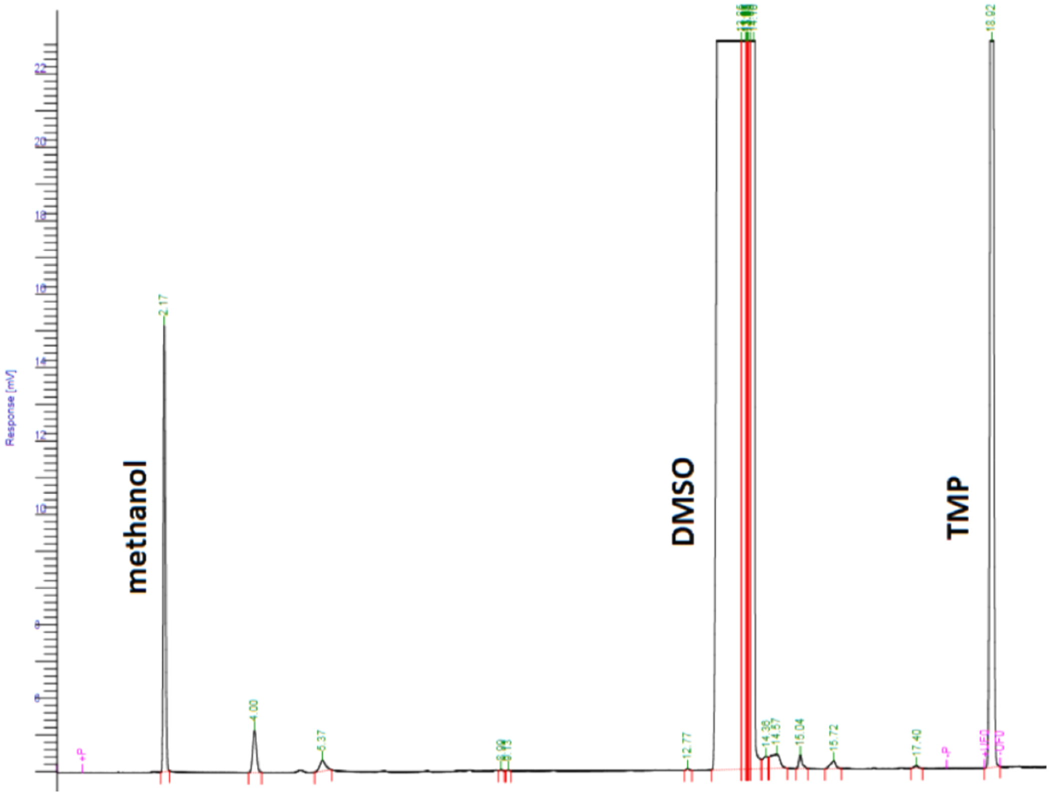

Figure S21. Gas chromatogram for **$[Ag_2(O_2CCF_2CF_2CO_2)(TMP)]\cdot(pentane)_x$** after dissolution in DMSO.

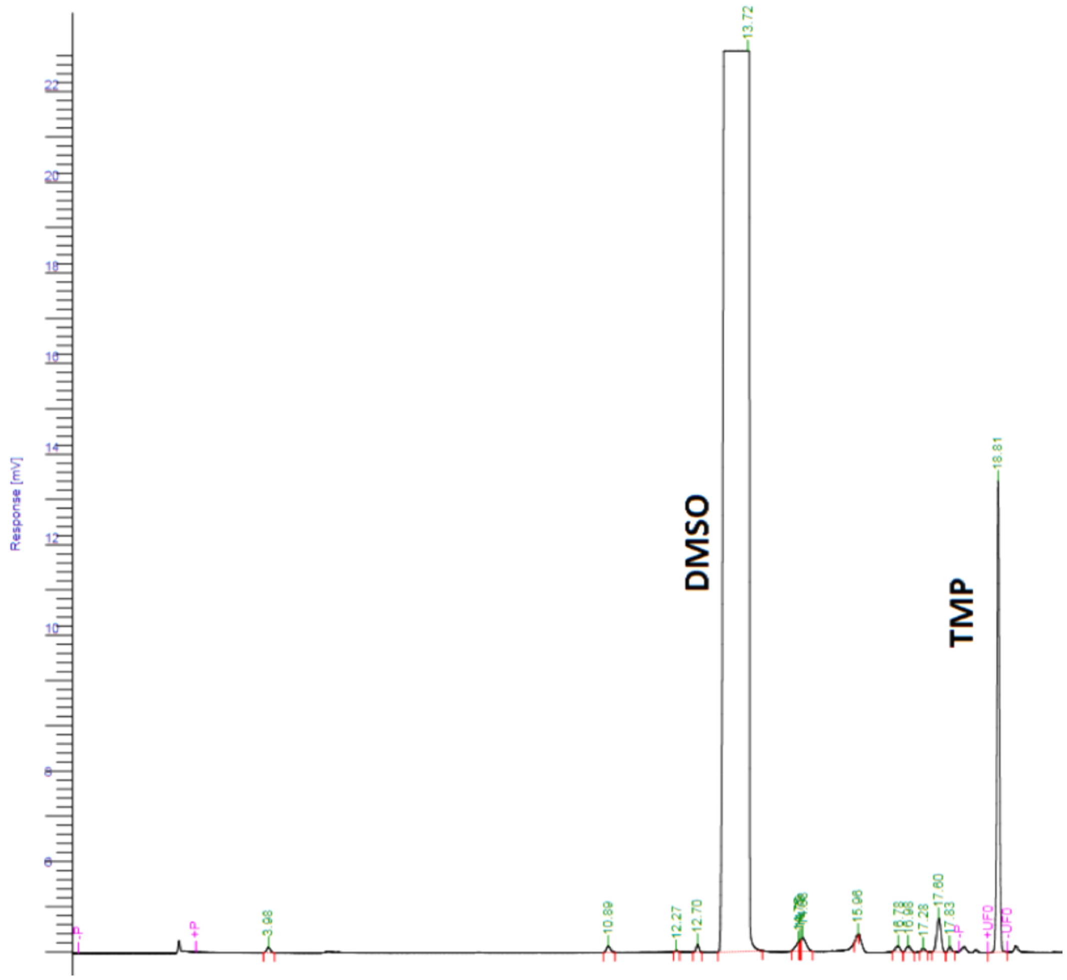

Figure S22. Gas chromatogram for **[$Ag_2(O_2CCF_2CF_2CO_2)$(TMP)]·(perfluoro-(methylcyclohexane))$_x$** after dissolution in DMSO.
